# Replicative *Acinetobacter baumannii* strains interfere with phagosomal maturation by modulating the vacuolar pH

**DOI:** 10.1371/journal.ppat.1011173

**Published:** 2023-06-09

**Authors:** Jesus S. Distel, Gisela Di Venanzio, Joseph J. Mackel, David A. Rosen, Mario F. Feldman

**Affiliations:** 1 Department of Molecular Microbiology, Washington University School of Medicine, Saint Louis, Missouri, United States of America; 2 Department of Pediatrics, Division of Infectious Diseases, Washington University School of Medicine, Saint Louis, Missouri, United States of America; Purdue University, UNITED STATES

## Abstract

Bacterial pneumonia is a common infection of the lower respiratory tract that can afflict patients of all ages. Multidrug-resistant strains of *Acinetobacter baumannii* are increasingly responsible for causing nosocomial pneumonias, thus posing an urgent threat. Alveolar macrophages play a critical role in overcoming respiratory infections caused by this pathogen. Recently, we and others have shown that new clinical isolates of *A*. *baumannii*, but not the common lab strain ATCC 19606 (19606), can persist and replicate in macrophages within spacious vacuoles that we called *A**cinetobacter* Containing Vacuoles (ACV). In this work, we demonstrate that the modern *A*. *baumannii* clinical isolate 398, but not the lab strain 19606, can infect alveolar macrophages and produce ACVs *in vivo* in a murine pneumonia model. Both strains initially interact with the macrophage endocytic pathway, as indicated by EEA1 and LAMP1 markers; however, the fate of these strains diverges at a later stage. While 19606 is eliminated in an autophagy pathway, 398 replicates in ACVs and are not degraded. We show that 398 reverts the natural acidification of the phagosome by secreting large amounts of ammonia, a by-product of amino acid catabolism. We propose that this ability to survive within macrophages may be critical for the persistence of clinical *A*. *baumannii* isolates in the lung during a respiratory infection.

## Author summary

*Acinetobacter baumannii* is an important nosocomial pathogen with the highest rate of multi-drug resistance among Gram negative bacteria. Although *A*. *baumannii* is capable to cause multiple types of infection, pneumonia is the main manifestation of this pathogen. We have recently shown that recent *A*. *baumannii* clinical isolates are able to multiply and persist in macrophages in vitro. Here we show that in a murine respiratory infection model, *A*. *baumannii* can also create niches within resident macrophages of the lung. Furthermore, we demonstrate that *A*. *baumannii* manipulates the macrophage endocytic pathway to create a replicative niche. Recent clinical isolates can withstand the acidic environment on the vacuole and neutralize it by secreting ammonia to the vacuolar lumen, which allows *A*. *baumannii* to further multiply inside the macrophage. The ability to survive and multiply within macrophages could be important to avoid the host immune system, avoid clearance, and withstand antibiotic treatment as many antibiotics cannot penetrate host cells.

## Introduction

The opportunistic bacterial pathogen *Acinetobacter baumannii* is an urgent global public health threat. This pathogen is associated with a wide variety of nosocomial infections including pneumonia, meningitis, urinary tract infection, and wound infection. However, respiratory tract infections are the principal disease associated with *A*. *baumannii* [1]. Alveolar macrophages (AMs) and lung epithelial cells are the first line of defense against respiratory pathogens [2,3]. Macrophages are particularly important during the early stages of *A*. *baumannii* respiratory infection, as they initiate and regulate the innate immune response against *A*. *baumannii* by recruiting non-resident macrophages and neutrophils [4–6]. Consequently, depletion of AMs during *A*. *baumannii* infection results in an increased in bacterial burden, tissue damage, and sepsis in the host [7]. Moreover, it has been demonstrated that murine AMs can internalize *A*. *baumannii in vivo* at 4 h post intranasal infection [5].

Phagocytosis is a mechanism by which cells internalize large particles, such as apoptotic cells or bacteria. Phagocytosis is required to maintain cellular homeostasis and is essential in the innate immune response against pathogens [8,9]. This process is performed primarily by myeloid cells including macrophages, neutrophils, monocytes, and dendritic cells [10,11]. After particle recognition and internalization, the newly formed phagosome undergoes a dynamic series of steps called “maturation”. Each phase of the process is determined by the presence of specific signaling molecules located on the phagosomal membrane. Initially, the phagosome recruits the characteristic endosomal marker Rab5 with its effectors EEA1 and VPS34. Maturation continues with the exchange of these markers by late endosome-associated molecules, such as Rab7, HOPS complex, LAMP1 and LAMP2 among others [12–14]. The process concludes with the formation of the phagolysosome and the degradation of its internal content by the activity of proteases, nucleases and lipases [15–17]. During maturation phagosomes progressively decrease luminal pH, mainly through proton pump Vacuolar ATPases (V-ATPase) activity [18,19]. This acidification is critical to halt growth of pathogenic microorganisms and is necessary for diverse microbicidal functions of the phagosome that depend on H^+^ concentrations, such as activation of degradative enzymes and production of reactive oxygen species (ROS) [20,21].

Many pathogenic bacteria have developed various strategies to hijack the host machinery, avoid phagosome maturation, and produce a specific compartment where intracellular bacteria can survive and replicate [22,23]. *Brucella abortus*, *Legionella pneumophila* and *Chlamydia trachomatis* escape early from the endocytic pathway and produce unique vacuoles that contain endoplasmic reticulum and golgi membrane markers [24–26]. *Mycobacterium tuberculosis* and *Salmonella* block phagosome maturation and arrest the bacterial-containing vacuole at an early stage [27–29]. Alternatively, *Coxiella burnetii* interacts with the endocytic pathway to develop a replication-permissive spacious vacuole that is similar to a phagolysosome [30,31]. *A*. *baumannii*, on the other hand, has classically been considered an extracellular pathogen. Recent reports, however, have demonstrated that *A*. *baumannii* clinical isolates, but not “domesticated lab strains”, can replicate *in vitro* within *Acinetobacter* Containing Vacuoles (ACVs) in lung epithelial cells and macrophages[32,33]. The trafficking pathways involved in the biogenesis of these ACV remains unknown.

In this work, we demonstrate that the modern clinical isolate of *A*. *baumannii*, 398, can survive and replicate inside AM ACVs during a murine pulmonary infection. Moreover, we characterize the intracellular trafficking of *A*. *baumannii* and describe key differences between the “domesticated lab strain” 19606 and the new clinical isolate 398 in J774A.1 macrophages. We additionally demonstrate that the replicative strain (strains that can replicate *in vitro* in eukaryotic cells) generates a non-degradative ACV. Finally, we provide evidence that resistance to acidic pH and secretion of ammonia, a by-product of amino acid degradation, are key factors for the *A*. *baumannii* intracellular survival.

## Results

### The *A*. *baumannii* recent clinical isolate 398 develops ACVs in alveolar macrophages *in vivo*

**Fig 1:**
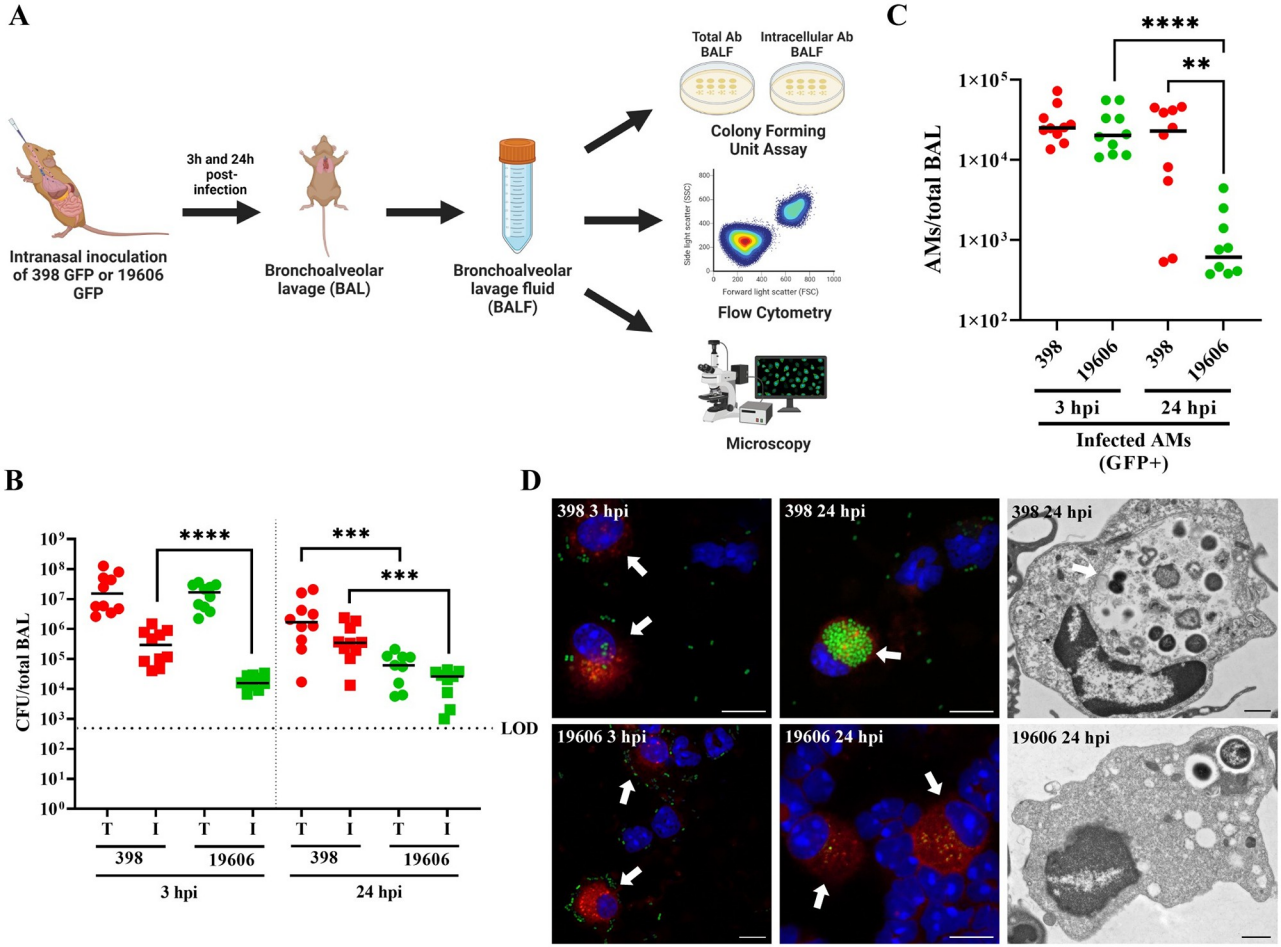
*A*. *baumannii* clinical isolate 398 infects AMs *in vivo* and survives inside ACVs. (A) Schematic of the pneumonia model employed in this study. (B) Mice were intranasally infected with 1 x 10^8^ CFU of *A*. *baumannii* strains GFP-19606 or GFP-398. At 3 or 24 hpi the total (T) or intracellular (I) CFU from bronchoalveolar lavages were determined. Symbols represents individual animals. Median values are shown as horizontal black bars. Data from two independent experiments with 5 mice per experimental group are shown. (C) Quantification of infected AMs (CD45+CD11c+SiglecF+CD11b-GFP+) obtained from BALF at 3 hpi or 24 hpi with GFP-19606 or GFP-398 strains. Symbols represent individual animals. (D) Representative confocal microscopy images of cells present in the BALF of infected mice (left and central images). Cell nuclei were stained with DAPI (blue), *A baumannii* 19606 or 398 were detected by GFP fluorescence (green), and SiglecF was immunolabeled with specific antibodies (red). Arrows indicate AMs. Scale bars: 10 μm. Representative transmission electron microscopy images with infected AMs from BALF are shown at the right. The arrow indicates 398 ACV. Scale bars: 1 μm. Statistical analyses were performed using the Mann–Whitney test, **p < 0,0021, ***p<0,0006 ****p < 0,0001. Hpi: hours post-infection.

Macrophages play a critical role in host immune defense against *A*. *baumannii* respiratory infections [5]. It has been reported that macrophages can phagocytose and degrade common lab strains of *A*. *baumannii* such as 19606, but diverse new clinical isolates persist and/or replicate in mouse and human macrophages [33,34]. We have previously demonstrated that *A*. *baumannii* new clinical isolates replicate in macrophages inside an *Acinetobacter* Containing Vacuole (ACV) [33]. Yet, to the best of our knowledge, there is no evidence showing that *A*. *baumannii* replicates inside macrophages *in vivo*. To analyze that, we evaluated the ability of two *A*. *baumannii* strains, the “domesticated lab strain” 19606 and the recent clinical isolate 398, to produce ACVs in macrophages during an acute lower respiratory tract infection into C57BL/6 mice. Both *A*. *baumannii* strains expressing GFP were each inoculated intranasally in C57BL/6 mice (∼1 × 10^8^ CFU), and after 3 and 24 hpi, the number of total and intracellular colony forming units (CFUs) present in the bronchoalveolar lavage fluid (BALF) was determined by antibiotic protection assays. Additionally, the number of total and infected AMs were analyzed using flow cytometry. Finally, the presence of ACVs in macrophages was established by confocal microscopy (Fig 1A). CFU enumeration demonstrated similar numbers of total bacteria for both *A*. *baumannii* strains at 3 hpi, indicating that similar numbers of bacteria reached the lung (S1A Fig). However, the number of intracellular bacteria was greater for 398 than for 19606 (Fig 1B, left). At 24 hpi, both the total and intracellular CFUs for 398 were significantly higher compared to 19606 (Fig 1B right). Similar results were found in the rest of the lung (S1A Fig). In agreement with Qiu et al., (2012) [5], we detected an slight increase in the number of AMs between 3 hpi and 24 hpi by flow cytometry, however at 24 hpi neutrophils were the most prevalent immune cells (S1B Fig). The total number of AMs present in the BALF was similar between mice infected with both *A*. *baumannii* strains, but the number of AMs infected with 398 persisted over time, while the ones infected with 19606 significantly decreased at 24 hpi (Fig 1C). Visualization of the cells present in the BALF by confocal and transmission electron microscopy (TEM) showed that only 398 was able to produce large ACVs in AMs, while the “domesticated lab strain” 19606 remained contained in single-bacterium phagosomes or was degraded by 24 hpi (Figs 1D and S1C). Moreover, large 398 ACVs (vacuoles containing more than ~10 bacteria) were found in more than 40% of the infected AMs, whereas no big vacuoles containing 19606 were observed at 24 hpi (S2 Fig). Together, these experiments demonstrate that, while a lab strain is rapidly cleared, the clinical isolate 398 can infect, persist and possibly multiply intracellularly inside ACVs in murine AMs *in vivo*.

### ACVs mature to a late phagosome

We have recently shown that some recent *Acinetobacter* strain are able to replicate inside ACVs in macrophages while other strains, including lab-domesticated strain 19606 are quickly eliminated by the host cell. To further validate our published results, we quantified “replicative centers” for 398 in J774A.1 macrophages at different times pi (S3 Fig). We define replicative centers as ACVs with a size three times larger than the median size of the vacuoles at 2 hpi. Strain 398 is able to form replicative centers at 4, 6 and 24 hpi, while 19606 vacuoles have the same size during infection and are absent at 24 hpi. Thus, 398 will be used for this study as a “replicative strain” which is able to replicate in macrophages *in vitro* [33] and 19606 as a “non-replicative strain”.

**Fig 2:**
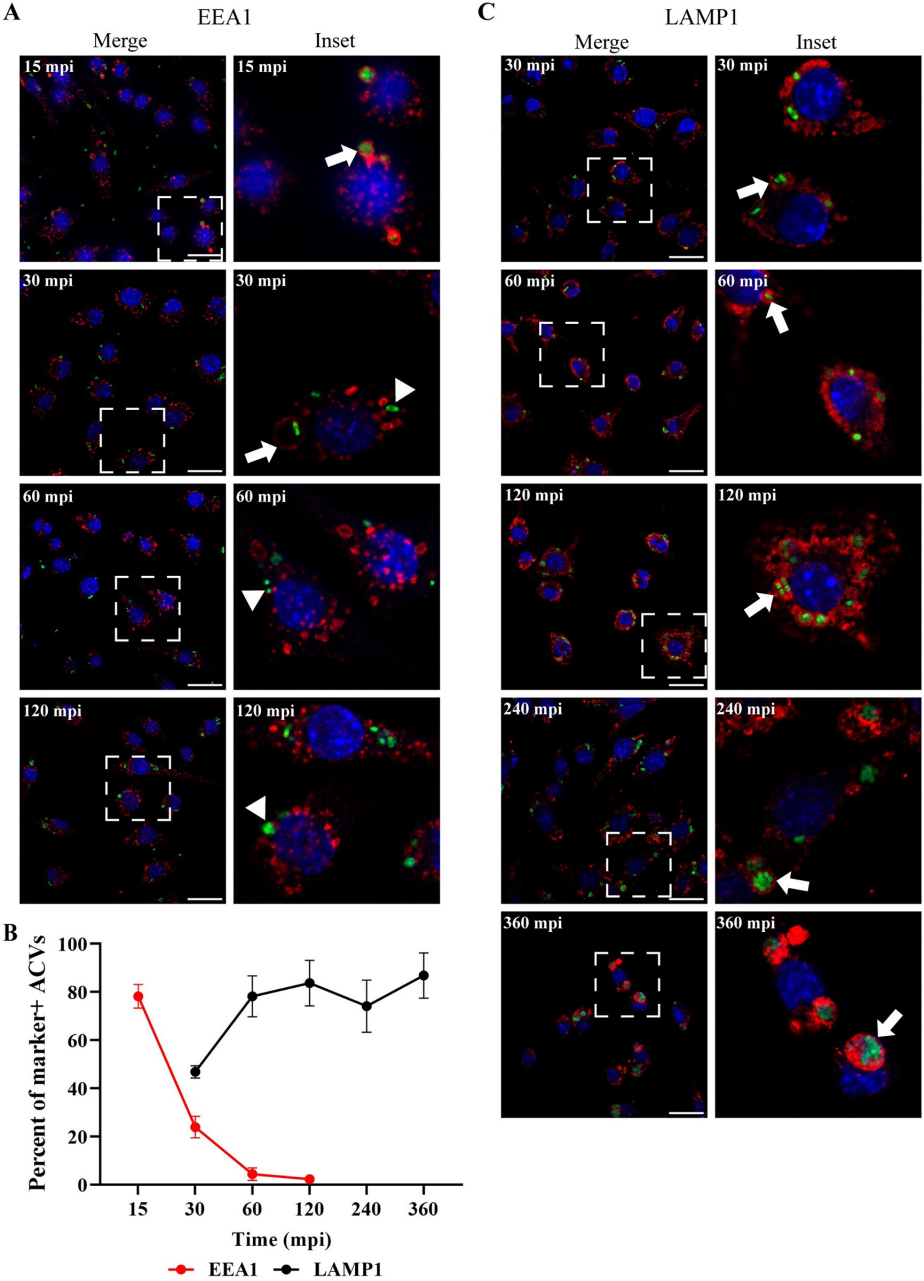
The ACV interacts with the endocytic pathway. J774A.1 cells were infected with *A*. *baumannii* GFP-398, and after the indicated times pi the cells were fixed and processed for confocal microscopy. The samples were stained to detect cell nuclei (blue), GFP-bacteria (green) and (A) EEA1 (red) or (C) LAMP1 (red). Bars: 20 μm. Insets (40 μm) are a higher magnification of the region indicated with a white box in the corresponding image. The presence of EEA1 or LAMP1 markers in the ACV is indicated by arrows while marker negative ACVs are denoted by arrowheads. (B) The percent of EEA1 or LAMP1 positive ACVs was determined at different times pi. At least 200 infected cells were analyzed per indicated time point. The results are expressed as mean ± SEM of three independent experiments. Mpi: minutes post-infection.

Nascent phagosomes interact with different compartments of the endocytic pathway to ultimately fuse with lysosomes and degrade their internal content. This sequential process is called “maturation” and is characterized by the recruitment of specific molecular markers to the phagosomal membrane that guide their traffic inside the cell [14]. However, several bacterial pathogens have evolved to manipulate the host cell and survive intracellularly [22,23]. To characterize the biogenesis of the ACVs, we utilized the early or late endosomal markers EEA1 (Early Endosomal Antigen 1) and LAMP1 (Lysosomal-associated membrane protein 1), respectively. Approximately, 80% of the ACVs of *A*. *baumannii* clinical isolate 398 colocalized with EEA1 as early as 15 min post infection (mpi), however, the percentage of colocalization of this marker decreased over time. At 60 mpi less than 5% of the ACVs were EEA1 positive (Figs 2A, 2B and S4). When we analyzed the distribution of the late endosomal marker LAMP1, most of the ACVs were decorated with this marker as early as 60 mpi (Figs 2B, 2C and S5A). Similar results were obtained with 19606 for LAMP1 endosomal marker (S5B and S5C Fig). Interestingly, no differences in cytotoxicity levels, as measured by the release of the cytoplasmic enzyme lactate dehydrogenase (LDH) to the extracellular media, were detected in cells infected with 398 or 19606 (S6 Fig). This data demonstrates that in macrophages the phagosomes produced by both strains initially follow a canonical maturation.

### The autophagic protein LC3 is absent from ACVs of the replicative *A*. *baumannii* 398 strain

**Fig 3:**
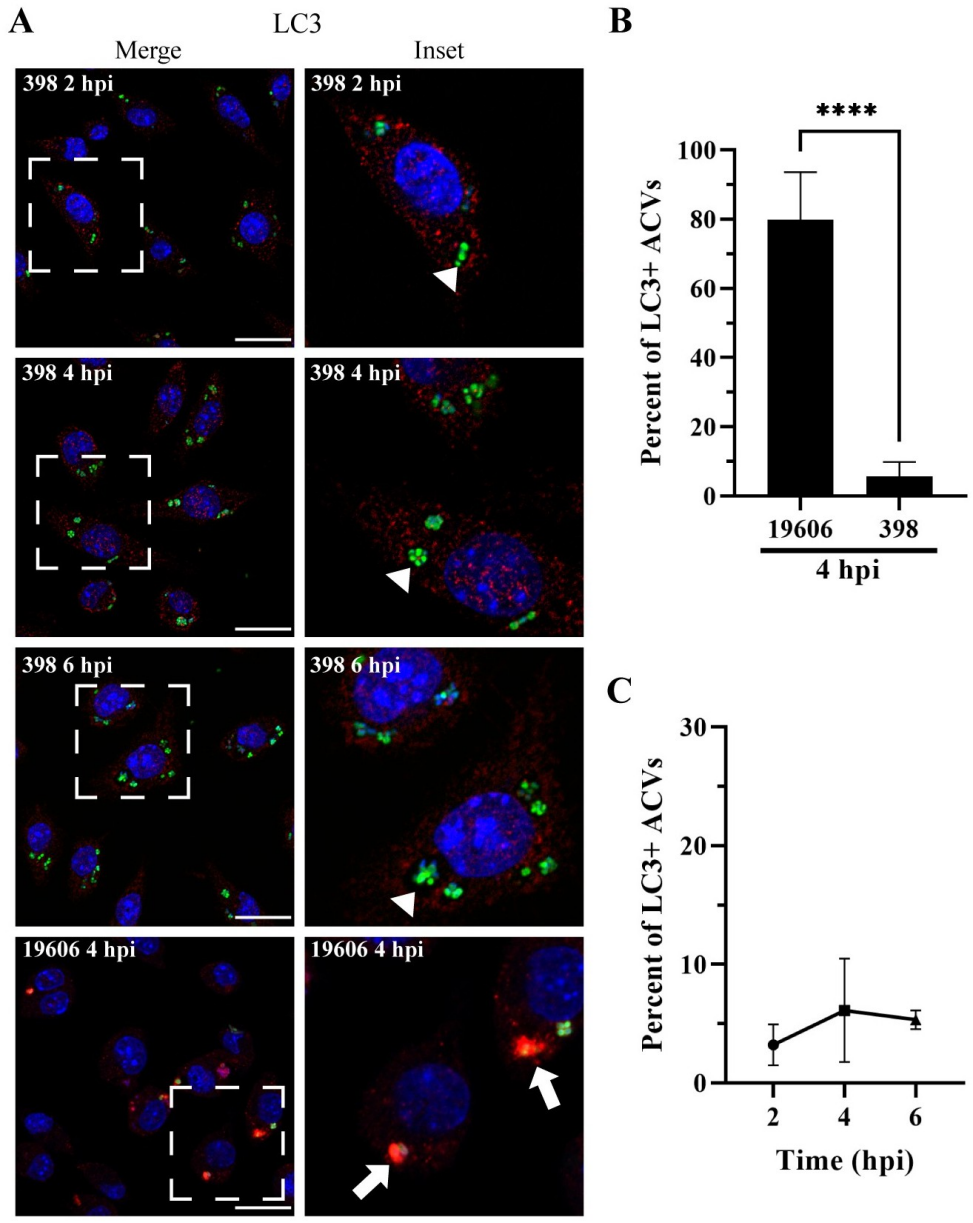
398 ACV does not colocalize with the autophagic marker LC3. (A) Representative confocal microscopy micrographs of J774A.1 cells infected with *A*. *baumannii* strains GFP-398 or GFP-19606 at the different times post-infection (pi). Cell nuclei were stained with DAPI (blue), bacteria overexpress GFP (green) and LC3 was immunostained with a specific antibody (red). Scale bars: 20 μm. Insets (40 μm) are a higher magnification of the area indicated with a white box in the corresponding image. LC3-positive ACVs are indicated with arrows and LC3–negative ACVs are indicated with arrowheads. (B) Comparison of the percent of LC3 positive ACVs of 398 and 19606 at 4 hpi. The 398 data is the same as shown if panel C. (C) Quantification of LC3 positive ACVs of the clinical isolate 398 at the different times pi. At least 200 infected cells were analyzed per strain per time point. The results are expressed as mean ± standard error of the mean (SEM) of three independent experiments. Statistical analyses were performed using Welch’s t-test, **** < 0.0001.

Autophagy is a process by which eukaryotic cells remove unwanted material from the cell cytosol [35]. Xenophagy is a highly selective type of autophagy used by the host cells to detect and eliminate intracellular pathogens [36,37]. Bacterial phagosomes can recruit the canonical autophagy marker, LC3 (microtubule-associated protein 1 light chain 3), inducing the compartment maturation and content degradation. However, the relationship between autophagy and intracellular microorganisms is complex [38]. Some intracellular bacteria as *C*. *burnetii* and *Serratia marcescens* survive in an autophagic vacuole while others, such as *M*. *tuberculosis* and *L*. *pneumophila*, avoid the interaction with autophagosomes [39–43]. However, the relationship between the autophagic pathway and *A*. *baumannii* remains unclear and controversial. In epithelial cells, vacuoles of the strain AB5075 colocalize with LC3 before bacterial killing; however, ACVs of replicative strains C4 or ABC141 do not recruit the autophagic marker [32,44]. In our previous work we demonstrated that, in J774A.1 macrophages, ACVs of the clinical isolate UPAB1 are LC3 negative [33]. We hypothesized that these differences could depend on the *A*. *baumannii* strain used in these studies. To corroborate this, the subcellular distribution of LC3 was analyzed in J774A.1 cells infected with our replicative isolate GFP-398 or the non-replicative GFP-19606. At 4 hpi, most of 19606 phagosomes were labeled with the autophagic marker (Fig 3A bottom row, Figs 3B and S7). On the contrary, 398 ACVs were LC3 negative up to 6 hpi (Figs 3A and 3C and S7). These data suggest that while non-replicative strains such as 19606 are eliminated by autophagy, replicative strains like 398 avoid the interaction with the autophagic pathway.

### The ACV of *A*. *baumannii* strain 398 is a non-degradative compartment

**Fig 4:**
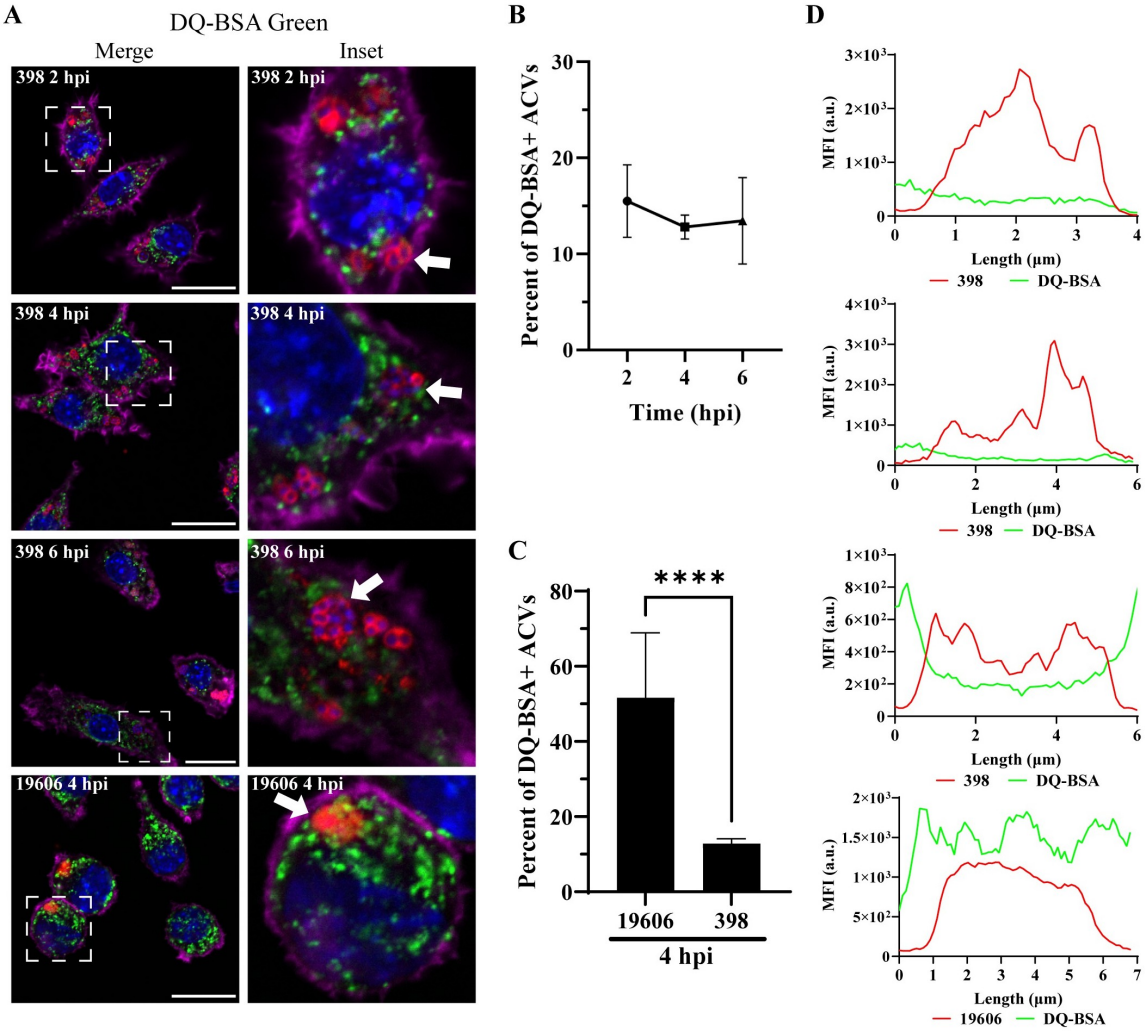
*A*. *baumannii* 398, but not 19606, resides in a non-degradative ACV. (A) J774A.1 cells infected with *A*. *baumannii* 398 or 19606 were incubated with DQ-BSA and then fixed at the indicated time points. The samples were stained to detect cell nuclei (blue), DQ-BSA (green), *A*. *baumannii* (red) and actin (pink). Bars: 20 μm. Insets (20 μm) are a higher magnification of the area denoted in the corresponding image with a white box. (B) Quantification of DQ-BSA positive ACVs of the clinical isolate 398 at the different times pi. (C) Comparison of the percentage of DQ-BSA positive ACVs of 398 and 19606 at 4 hpi. The 398 data is the same as shown if panel B. (D) Fluorescent intensity plots of representative ACVs (indicated with arrows in panel A). Statistical analyses were performed using the Welch’s t-test, **** < 0.0001. At least 200 infected cells were analyzed per strain, per time point. Results are expressed as mean ± SEM of three independent experiments.

The final step of phagosomal maturation is the fusion with lysosomes to form the phagolysosomal compartment in which the degradation of the internal content takes place [16]. To determine if the ACV is a degradative compartment, we used the fluorogenic probe DQ-BSA Green, a self-quenched substrate that fluoresces green when it is degraded by acidic proteases in the phagolysosome. The lysosomes of both uninfected and infected macrophages exhibited green fluorescence, indicating that lysosomal biogenesis and functions were not impaired overall (S8A and S8B Fig). More than 50% of 19606 phagosomes were degradative compartments (DQ-BSA positives) (Figs 4A, 4C and S8B bottom row). In contrast, more than 80% of 398 ACVs did not colocalize with the fluorogenic probe (Figs 4A, 4B, 4C and S8B). Similarly, the median fluorescence intensity (MFI) profiles of representative ACVs show that only phagosomes containing 19606 exhibit a high green signal corresponding to the degraded DQ-BSA marker (Fig 4D). These data indicate that the 398 ACVs are non-degradative, suggesting that 398 can manipulate the canonical maturation of the phagosome.

### Luminal pH of the ACV increases during infection

**Fig 5:**
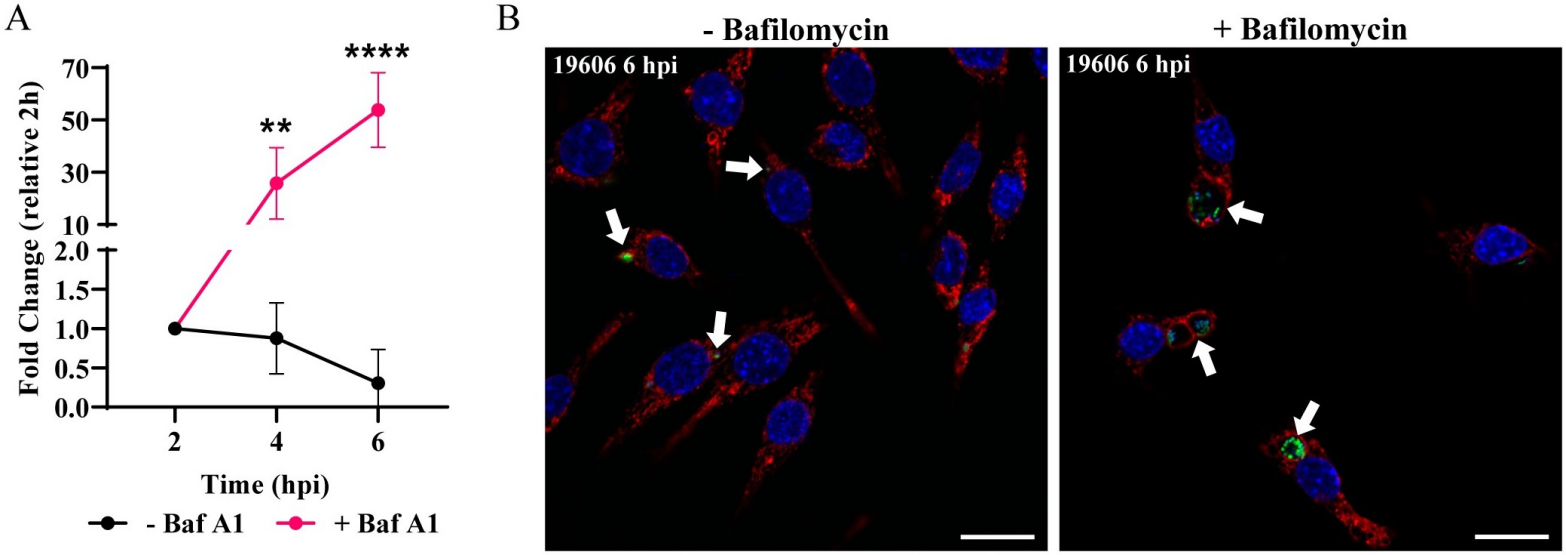
Bafilomycin A1 treatment allows 19606 to replicate in macrophages. (A) J774A.1 macrophages were infected with GFP-19606 and treated with the proton pump V-ATPase inhibitor bafilomycin A1. Total numbers of intracellular CFU were determined at different times pi in treated and non-treated cells. Statistical analyses were performed by two-way ANOVA-test, ** < 0.0021, **** < 0.0001. (B) Representative images of cells infected with GFP-19606 (green) and incubated with or without bafilomycin A1 at 6 hpi are shown. Cell nuclei were stained with DAPI (blue) and LAMP1 with specific antibody (red). Bars: 20 μm.

The two main characteristics of phagolysosomes are an acidic pH and the presence of hydrolytic enzymes [8]. Phagosome acidification is mainly achieved by V-ATPases that pump H^+^ into the luminal space of this compartment. To test the effect of vacuolar acidification on *A*. *baumannii* intracellular replication, we used bafilomycin A1, a specific V-ATPase inhibitor. While strain 19606 was degraded under normal conditions, in the presence of bafilomycin A1, 19606 was able to replicate (Fig 5A). Moreover, large ACVs with many GFP-19606 bacteria were observed by confocal microscopy when incubated with bafilomycin A1 (Fig 5B). Furthermore, 398 intracellular replication was higher in the presence of bafilomycin A1 (S9 Fig). These data suggest that the luminal pH of the ACV is key factor in thwarting intracellular replication of *A*. *baumannii*.

**Fig 6:**
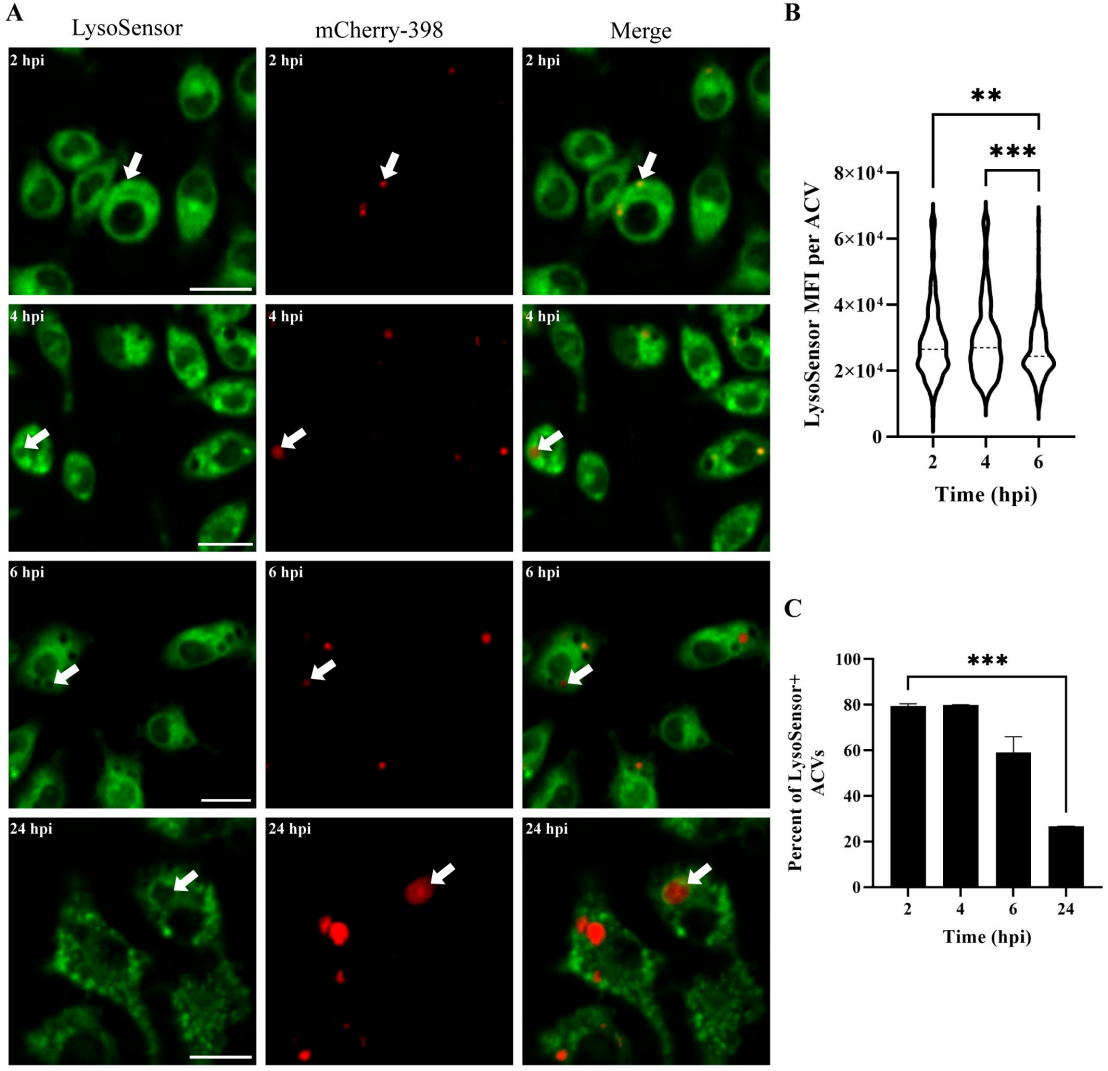
398 increases the luminal pH of the ACV during infection. (A) J774A.1 cells infected with mCherry-398 (red) were incubated with LysoSensor (green) 15 minutes before the indicated time points. Samples were analyzed by *in vivo* confocal microscopy. Bars: 20 μm. (B) Analysis of the Mean Fluorescence Intensity (MFI) signal of LysoSensor per ACV at different times pi. Dotted lines show the median. (C) Percentage of ACVs that colocalize with LysoSensor at 2, 4, 6 and 24 hpi. Statistical analyses were performed using one way ANOVA-test, ** < 0.0021, *** < 0.0002. At least 200 infected cells were analyzed per indicated time point. Results are expressed as mean ± SEM of three independent experiments.

The ability of 398 to survive and replicate within ACVs prompted us to analyze their pH. LysoSensor is a dye that is internalized in the cell, and its fluorescence intensity is dependent on the pH of the compartment in which it resides (increased brightness at low pH). Live confocal microscopy demonstrated the presence of LysoSensor (green) in the ACVs (red) early during infection for both 398 and 19606 ACVs (Fig 6A, 2 and 4 hpi white arrow and S10A Fig), indicating that most of the vacuoles were initially acidic. However, as 398 infection progresses and the bacteria replicate inside the vacuole, the lumen of the ACV decreases in green signal intensity (Fig 6A, 6 and 24 hpi white arrow), revealing a remarkable increase in the luminal pH of this compartment (Fig 6A and 6B). On the contrary, the green signal intensity of 19606 vacuoles are higher at all times pi, indicating an acidic vacuolar pH (S10B Fig). Moreover, the number of 19606 vacuoles that colocalize with LysoSensor increase significantly over time (S10C Fig) while it decreases for 398 ACVs (Fig 6C). Furthermore, no 19606 vacuoles can be detected at 24 hpi (S10A Fig, bottom row) suggesting the degradation of the intracellular bacteria. These results demonstrate that although 398 vacuoles are initially acidic this strain, but not 19606, can actively increase the ACV’s luminal pH.

### Intracellular replication relies on tolerance to acidic pH and ammonia production

**Fig 7:**
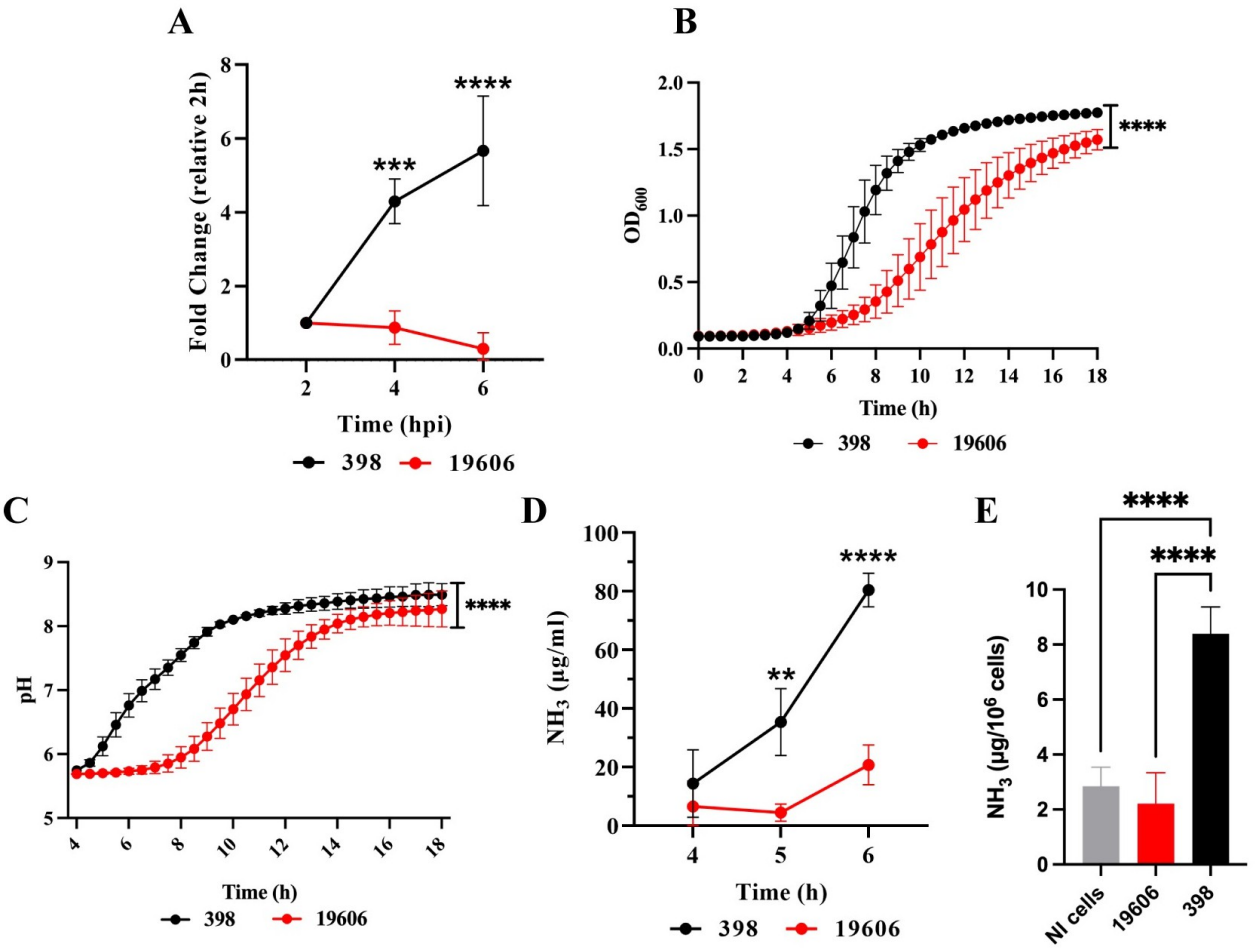
Intracellular replication correlates with the ability to grow at acidic pH. (A) The intracellular replication of *A*. *baumannii* strains 398 and 19606 in J774A.1 macrophages was determined by antibiotic protection assays. (B) Growth curves of 398 and 19606 in buffered LB pH 5 as measured by OD_600_. (C) Changes in culture pH during *A*. *baumannii* growth, determined by phenol red absorbance at 560 nm. (D) Concentration of ammonia in LB cultures of 398 and 19606 strains at 4, 5 and 6 h post-inoculation. Results are expressed as mean ± SEM of three independent experiments. (E) Concentration of ammonia per million cells in macrophages infected with 398 or 19606 strains at 24 hpi. Results are expressed as mean ± SD of three independent experiments. ****< 0.0001, ***< 0.001 and **< 0.01.

Our data suggests that the ACV pH is key in controlling *A*. *baumannii* replication and that the clinical isolate 398 actively increases the pH of the ACV after initially surviving acidic conditions. Previous work observed that *Acinetobacter calcoaceticus* clinical isolates are able to survive at acidic conditions (pH 4 to 6), but growth was significantly reduced compared to neutral pH [45]. Also, *A*. *baumannii* was found to be more tolerant to pH stress than other *Acinetobacter* species such as *A*. *nosocomialis*, *A*. *pittii* and *A*. *calcoaceticus* [46]. We hypothesized that replicative strains of *A*. *baumannii* are more tolerant to acid stress than non-replicative bacteria and as a result they can grow at faster rates under acidic conditions. To analyze the relationship between intracellular replication and growth fitness at acidic pH, we employed the replicative strain 398 and the non-replicative strains 19606 (Fig 7A). We tested the ability of these strains to grow in buffered LB broth at pH 5, 6, or 7, as previously described. Although the two strains grew similarly at neutral pH (S11 Fig), 19606 had a lower growth rate than 398 at pH 5 (Fig 7B). In non-buffered LB (pH ≈5), tested strains alkalinized the culture media to pH≈8 after growth for 18 h. However, 398 required significantly less time to neutralize the media than the non-replicative strain. For example, 398 reaches a pH of ≈7 at 6 h, while 19606 requires ≈10 h to reach the same pH (Fig 7C). *A*. *baumannii* is unable to grow using most sugars individually [47], instead, it heavily relies on amino acids. To feed the TCA cycle, the α-amino groups of the amino acids are removed with the concomitant production of ammonia, which is secreted to the media, resulting in an increase of the pH [48]. We measured the secretion of ammonia *in vitro* at pH ≈5 by the two strains and found that 398 produces significantly higher amounts of ammonia than 19606 (Fig 7D) at earlier time points. Furthermore, we detected higher levels of intracellular ammonia in macrophages infected with 398 compared to non-infected cells or cells infected with 19606 (Fig 7E). Employing additional strains, we observed that intracellular replication correlates with the ability of the strains to withstand acidic pH and to produce ammonia *in vitro* (S12 Fig). Together, our results show that tolerance to acidic pH and the subsequent production and secretion of ammonia to neutralize the intravacuolar pH are key for *A*. *baumannii* replication within the ACV.

## Discussion

**Fig 8:**
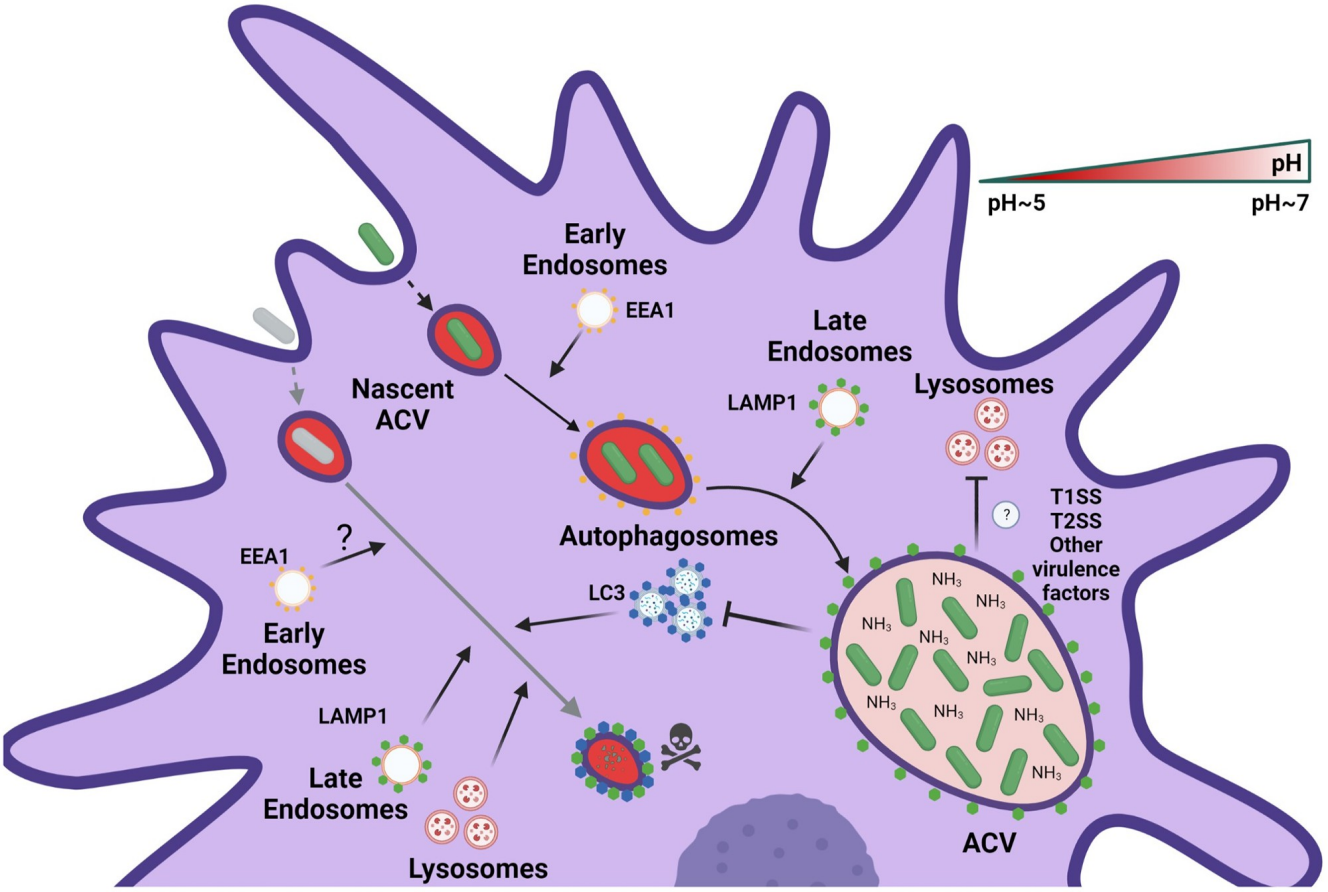
Proposed model of the intracellular lifestyle of *A*. *baumannii* in macrophages. Replicative strains of *A*. *baumannii* (green bacterium) interact with the endocytic pathway sequentially acquiring EEA1 and LAMP1 markers in the ACV. However, during its maturation, the ACV does not interact with autophagosomes or lysosomes of the host cell. Moreover, the replication of intracellular bacteria produces ammonia which neutralizes the luminal microenvironment of the ACV. On the other hand, non-replicative strains of *A*. *baumannii* (gray bacterium) are phagocytized by macrophages and reside in a nascent phagosome. This compartment maturates to a late phagosome, like the replicative ACV, by interactions with the endocytic pathway. Nevertheless, the final fate of the non-replicative strains of *A*. *baumannii* is the degradation in an autophagolysosome.

Here, we demonstrate that classic *A*. *baumannii* lab strains, like 19606, and recent clinical isolates, such as 398, behave differently, both *in vivo* and *in vitro*. While 398 was able to replicate in large ACVs within AMs during murine respiratory infection, 19606 was quickly cleared by these cells. Fig 8 summarizes our findings related to the intracellular behavior of both replicative and non-replicative strains *in vitro*. Initially, both strains follow the canonical endocytic pathway, as determined by the sequential colocalization of EEA1 and LAMP1 markers with phagosomes. 19606 phagosomes continue with their maturation and fuse with autophagosomes and lysosomes to ultimately be degraded by the macrophage. However, our data show that 398 ACVs avoid the autophagic and degradative pathways allowing the bacteria to survive and replicate inside macrophages. Moreover, 398’s ability to quickly grow at acidic pH allows this strain to revert the initial luminal acidification of the vacuole, most likely via production of ammonia, a by-product of amino acid degradation. The process generates a non-degradative niche that allows replication of *A*. *baumannii* inside the ACV.

Several reports have established the critical role of AMs during *A*. *baumannii* respiratory infections [5,7,49,50]. Depletion experiments demonstrated that AMs are necessary to control tissue damage, bacterial sepsis and severity of the infection in an *A*. *baumannii* pneumonia models [7]. Recent reports showed that *A*. *baumannii* modern clinical isolates are able to survive and/or replicate intracellularly, *in vitro*, in macrophages [33,34] and lung epithelial cells [32]. To the best of our knowledge, this is the first demonstration of the presence of large vacuoles containing *A*. *baumannii* within AMs in a mammalian pneumonia model. A similar behavior has been described for other pathogens, such as *M*. *tuberculosis*, *Staphylococcus aureus*, *and L*. *pneumophila*, in which bacteria use alveolar macrophages as a reservoir to persist and replicate within the host [51–54]. Therefore, replication in AMs could be a key factor for the persistence of *A*. *baumannii* within the infected hosts.

We demonstrated that the intracellular compartment produced by replicative *A*. *baumannii* strains such as 398 was clearly different than the classical phagosome formed by non-replicative strains such as 19606. Avoiding fusion with autophagosomes is critical for some intracellular bacteria to escape from degradation. The interaction between *A*. *baumannii* and autophagy has been studied in different host cells, but the conclusions were disparate. In epithelial cells, non-replicative *A*. *baumannii* strains induce autophagy, leading in some cases to persistence [55,56], or degradation [44,57]. In THP-1 monocytes or MH-S macrophages, induction of autophagy after infection with *A*. *baumannii* strain 98-37-09 leads to bacterial killing by the host cell [57]. Moreover, LC3 colocalized with *A*. *baumannii* strains AB5075 and 98-37-09 internalized by epithelial cells [44,57]. However, our data is in agreement with recent works that show that new clinical isolates that replicate inside ACVs in epithelial cells or macrophages do not recruit LC3 or get degraded [32,33].

Phagosomal acidification, produced mainly by the accumulation of V-ATPases, is critical to eliminate some bacteria [20]. Several intracellular pathogens have developed strategies to avoid phagosomal acidification [58,59]. Here, we observed that initially the ACV of the replicative isolate 398 is acidic; however, as the infection progresses, the strain can actively increase the internal pH of the compartment. Moreover, we determined that *A*. *baumannii* replicative strains can better withstand acidic conditions and grow to a faster rate than non-replicative strains. This allows these strains to release higher levels of ammonia, leading to a quicker neutralization of the vacuolar pH (Figs 7 and S12). While most prominent Gram-negative intracellular pathogens use a type III or IV secretion system [60,61] to manipulate the host cell machinery, *A*. *baumannii* only has a Type I secretion systems (T1SS) and Type II secretion systems (T2SS) [62]. Multiple reports have established that the presence of ammonia in the phagosome inhibits the fusion of this compartment with lysosomes in macrophages [63–65]. Other pathogens such as *Cryptococcus neoformans* or *Helicobacter pylori* can increase the phagolysosomal pH expressing a urease to produce ammonia [66,67]. *M*. *tuberculosis* secretes the asparaginase AnsA and avoids phagosomal acidification by the production of ammonia, enabling intracellular replication [68]. We propose a model in which *A*. *baumannii* strains that resist the initial acidic stress of the phagosome are able to grow and release ammonia as a metabolic by-product, thereby neutralizing the microenvironment of the ACV. Future work will determine if *A*. *baumannii* employs T1SS or T2SS effectors to aid in preventing acidification of the ACV and intracellular replication.

An increasing number of studies, including this one, demonstrate differences in intracellular behavior within macrophages among *A*. *baumannii* isolates [32–34,69,70]. Based on this evidence, we propose to redefine *A*. *baumannii* as a facultative intracellular bacterium that can grow outside and within the host cell [71]. From the results obtained in this work, it is evident that *A*. *baumannii* can develop ACVs in AMs *in vivo* in a murine pneumonia model and it is not a phenomenon restricted to *in vitro* observations. We recently demonstrated that the clinical isolate UPAB1 establishes small intracellular reservoirs, known as ABIR (*A*. *baumannii* intracellular reservoirs) in bladder epithelial cells of mice that previously cleared a urinary tract infection. We observed that these ABIRs likely act as reservoirs that can be activated upon insertion of a medical device, such a catheter, leading to a resurgent infection in mice [72]. It is tempting to speculate that intracellular *A*. *baumannii* in AMs may also function as reservoirs in the host. We hypothesize that this intracellular lifestyle of *A*. *baumannii* enhances persistence inside the host. Within AMs, *A*. *baumannii* is protected from the immune system and from antibiotics that do not penetrate eukaryotic cells, like aminoglycosides and polymyxins.

Here, we have demonstrated that *A*. *baumannii* strain 398 and possible some other recent clinical isolates, are able to manipulate the host intracellular environment to form a replicative niche. Future work will focus on the identification of additional factors that *A*. *baumannii* employs to survive inside these host cells, which may lead to improved therapeutic interventions to treat modern *A*. *baumannii* clinical isolates.

## Materials and methods

### Bacterial strains and culture conditions

The bacterial strains employed in this work are described in S1 Table. Bacteria were seeded on lysogeny broth (LB) agar plates and incubated 16 h at 37 °C. Thereafter, colonies were grown in LB broth under shaking conditions (200 rpm) for 16 h at 37 °C. When appropriate, antibiotics were added to the cultures at the following concentrations: 50 μg/ml zeocin, 30 μg/ml kanamycin, and 15 μg/ml chloramphenicol.

### Growth assays

*A*. *baumannii* strains were cultured overnight in LB broth at 37 °C under shaking conditions. Bacterial cultures were centrifugated at 6500 rpm for 5 min and the pellets were washed with Phosphate-buffered saline (PBS, Sigma, D8662). *A*. *baumannii* suspensions were diluted to OD_600_ = 0.01 in 150 μl of LB buffered at pH 5 (MES 100 mM), 6 (MES 100 mM), or 7 (HEPES 100 mM) in 96-well plates and incubated 16 h at 37 °C under shaking conditions. OD_600_ values were measured at 30 min intervals using a BioTek microplate spectrophotometer. The experiment was performed in biological triplicate with three technical replicates per experiment for each strain and condition.

### pH measurements in *A*. *baumannii* cultures

The absorbance of phenol red solutions at 560 nm is an indicator of pH, as previously described [73–75]. Phenol red is a pH indicator changing color from yellow below pH 6.8 to bright pink above pH 8.2. To analyze the pH changes in *A*. *baumannii* cultures during bacterial growth, indicated strains were maintained overnight in LB broth at 37 °C under shaking conditions. Bacterial cultures were washed and diluted as described for growth assays, with the following modification: non-buffered LB pH 5 with phenol red (15 μg/ml) was used. OD_600_ and OD_560_ values were measured every 30 min for 16 h using a BioTek microplate spectrophotometer. The OD_600_ absorbance values were subtracted from the absorbance at 560 nm data to exclude the effect of bacterial density. Finally, the values obtained were used to estimate culture pH by employing a standard curve of OD_560_ vs pH. The experiment was performed in triplicate with three technical replicates per each strain.

### Cell culture conditions

The J774A.1 mouse macrophage cell line (ATCC TIB-67) was cultured in Dulbecco’s Modified Eagle Medium (DMEM) High Glucose (Hyclone, SH30022.01) supplemented with 10% heat-inactivated Fetal Bovine Serum (FBS, Corning) at 37°C and 5% CO_2_.

### Murine Model of *A*. *baumannii* pneumonia

Colonies from *A*. *baumannii* strains 398, GFP-398 and GFP-19606 were subcultured from LB agar plates and grown in LB broth 16 h at 37°C under shaking conditions. Overnight cultures obtained were diluted in sterile LB broth (1:100) and incubated for 3 h at 37 °C under shaking conditions. The bacterial inocula were prepared by centrifugation at 6500 rpm for 5 min, washed twice in PBS, and the pellet resuspended in PBS. Six- to eight-week-old female C57BL/6 mice obtained from Charles River Laboratories were intranasally inoculated with *A*. *baumannii* strains 398, GFP-398, or GFP-19606. Briefly, mice were anesthetized by inhalation of 4% isoflurane and were infected immediately with 50 μL of bacterial suspension (∼1 × 10^8^ Colony Forming Units [CFU]) that were gradually released with a micropipette into the nares. At 3 and 24 hours post-infection (hpi), bronchoalveolar lavage fluid (BALF) was collected. For BALF collection, mice were euthanized by CO_2_ asphyxiation and then, the lungs and trachea were exposed by surgical dissection and polyethylene tubing (0.86x1.27mm) was inserted into the trachea. Cold PBS containing 1mM EDTA was flushed through the lungs in 1ml increments to collect a total volume of 10 ml. All pneumonia studies were performed in accordance with the guidelines of the Committee for Animal Studies at Washington University School of Medicine, and we have complied with all relevant ethical regulations.

### Antibiotic protection assay

To analyze the intracellular replication of the different strains, J774A.1 cells were seeded 16 h before the experiment in 48-well plates (3x10^5^ cells/well). *A*. *baumannii* colonies from LB agar plates were inoculated in LB broth and grown overnight at 37°C under shaking conditions. Bacterial cultures were centrifugated 5 min at 6500 rpm and washed twice with PBS. Then, bacterial cultures were normalized to OD_600_ ≈ 1 and appropriate volumes was added to 500 μl of DMEM per well to achieve an MOI≈10. Where indicated, bafilomycin A1 (15 nM; Sigma, B1793) was added to the growth media and maintained throughout the infection. Infected cells were centrifugated 10 min at 200 x g and incubated at 37°C and 5% CO_2_. After 1 h, macrophages were washed three times with PBS and treated with DMEM supplemented with 10% FBS and colistin (50 μg/mL) to eliminate the extracellular bacteria. At the time points indicated, the cells were washed three times with PBS and lysed with 500 μl of Triton X-100 (0.05%) per well. Serial dilutions of bacterial suspensions obtained were plated on LB agar and incubated overnight at 37 °C, to determine number of colony forming units (CFUs).

To determine the number of total and intracellular bacteria present in BALF from *A*. *baumannii* infected mice, two 500 μl aliquots of lavage fluid were centrifuged at 6500 rpm for 10 min. The pellets were incubated for 1 h at 37 °C in PBS (total bacteria) or PBS with colistin (50 mg/mL) (intracellular bacteria). Then the cells were washed three times with PBS and lysed with 500 μl of Triton X-100 (0.05%) per aliquot. CFUs were determined by serial dilutions of the bacterial suspensions.

### Ammonia production

Ammonia production by *A*. *baumannii* strains was measured using the Ammonia Assay Kit (AA0100, Sigma) according to the manufacturer’s instructions. Briefly, the strains were cultured overnight in LB broth at 37 °C under shaking conditions. Then, bacterial cultures were centrifugated, and washed with PBS. *A*. *baumannii* suspensions were diluted to OD_600_ = 0.01 in 150 μl of buffered LB (MES 100 mM, pH 5) in 96 well plates and incubated 6 h at 37 °C under shaking conditions. Ammonia levels were measured, at the indicated times. The experiment was performed in biological triplicate.

Intracellular ammonia production was measured using the same kit. Briefly, macrophages were infected with *A*. *baumannii* strains 19606 or 398 in duplicates. 24 hpi a set of wells was scraped in PBS to determine the number of macrophages. The remaining wells were treated with 200 μl of Triton X-100 (0.05%), centrifuged at 6500 rpm for 10 min and ammonia levels were measured in the supernatant.

### Flow cytometry

BALF samples were centrifugated at 300 x g for 5 min and incubated in 500 μl Pharm Lyse Buffer (BD Biosciences 555899) for 3 min at room temperature to lyse red blood cells. The cells were then washed with flow cytometry buffer [PBS + 0.5% Bovine Serum Albumin (BSA) (Fisher BioReagents, BP9706100) + 2 mM EDTA] and incubated at 4°C with Fc Block (BD Biosciences, 553142) for 10 min. Samples were then stained for 30 min with CD45-BV510 (Biolegend, 103138) and Ly6G-BV421 (Biolegend,127628), CD11b-Alexa700 (BD Biosciences, 557960), CD11c-APC (BD Biosciences, 550261), and Siglec-F-BV786 (BD Biosciences, 740956). Finally, cells were washed and fixed in 2% paraformaldehyde until acquisition on a Benton Dickinson (BD) LSR II Fortessa cytometer. GFP signal was acquired in the FITC channel. Total cell counts per mouse were calculated using Precision Count Beads (Biolegend, 424902) according to the manufacturer’s instructions.

### Cytospin of BALF cells

BALF samples were centrifugated at 300 x g for 5 min and the pellets were resuspended in 500 μl Pharm Lyse Buffer (BD Biosciences, 555899) for 3 min at room temperature to lyse red blood cells. The cells were resuspended in PBS and total cells and viability was determined using Trypan Blue solution (Sigma, T8154) and counted using the TC20 automated cell counter (BioRad). Samples were centrifugated at 300 x g for 5 min onto CytoPro Poly-L-Lysine Coated Microscope Slides (ELITechGroup, SS-118). The slides were air-dried overnight at 4 °C and fixed in 4% paraformaldehyde. Samples were incubated with permeabilizing and blocking solution [PBS + 0.1% saponin + 0.5% BSA (Fisher BioReagents, BP9706100) + 10% FBS (Corning)]. Cells were stained with Alexa Fluor 647 Rat Anti-Mouse Siglec-F antibody (BD, 562680) and nuclei with 4’,6-Diamidino-2-Phenylindole, Dihydrochloride (DAPI) solution (Invitrogen, D1306) 1 h at 37°C. After staining, the samples were rinsed with washing solution [PBS + 0.1% saponin + 0.5% BSA (Fisher BioReagents, BP9706100)], then rinsed with water, and mounted with a coverslip in Invitrogen ProLong Gold Antifade Mountant (Invitrogen, P36930). Finally, the cells were analyzed by confocal microscopy.

### Immunofluorescence staining

1.3x10^5^ J774A.1 cells were plated onto glass coverslips in 24 well plates and incubated 16 to 18 h at 37 °C and 5% CO_2_. Inocula of the indicated *A*. *baumannii* strains were prepared by centrifugation of overnight cultures at 6500 rpm for 5 min, washing twice in PBS, and resuspension of the pellet in PBS. Bacterial suspensions were normalized to OD≈1 and an appropriate volume was used to infect the cells (MOI≈10). Afterward, the plates were centrifugated 10 min at 200 x g to enhance bacterial contact with the host cells and incubated for 1 h at 37 °C and 5% CO_2_. Cells were washed three times with PBS, and extracellular bacteria were killed by treatment of the cells with colistin (50 μg/mL) for 1 h. When necessary, cells were incubated with DQ Green BSA (10 μg/ml; Invitrogen, D12050) 1 h prior to the end of the infection. At the indicated time points, samples were fixed with 4% paraformaldehyde for 15 min at 37 °C and then stored in permeabilizing and blocking solution [PBS + 0.1% saponin + 0.5% BSA (Fisher BioReagents, BP9706100) + 10% FBS (Corning)]. The glass coverslips were incubated with the indicated primary antibodies produced in rabbit: anti-LC3 (Sigma, L7543), anti-EEA1 (Invitrogen, PA1-063A), anti-LAMP1 (Abcam, ab24170) or anti-*A*. *baumannii* at a 1:100 dilution for 1 h at 37 °C. The cells were then washed 3 times with washing solution and incubated with the indicated secondary antibody goat anti-rabbit: Alexa Fluor 647 (Invitrogen, A-21244) at a 1:250 dilution, Alexa Fluor 555 phalloidin (0.33 μM; CST, #8953) and DAPI for 1 h at 37 °C. Afterwards, samples were washed with PBS, rinsed with water, and mounted with a coverslip in Invitrogen ProLong Gold Antifade Mountant (Invitrogen, P36930). Stained samples were analyzed by confocal microscopy.

### Confocal microscopy

Infected cells were analyzed with a Zeiss LSM880 laser scanning confocal microscope (Carl Zeiss Inc.) equipped with 405nm diode, 488nm Argon, 543nm HeNe, and 633nm HeNe lasers. A Plan-Apochromat 63X (NA 1.4) DIC objective and ZEN black 2.1 SP3 software were used for image acquisition. Live images were acquired with a Zeiss spinning disk confocal microscope (Carl Zeiss Inc.) equipped with 488nm and 560nm lasers. A Plan-Apochromat 63X/1.3 Oil Ph 3 (UV) VIS-IR M27 objective and ZEN black 2.1 SP3 software were employed for image acquisition. Images were analyzed using ImageJ software (NIH, USA).

### Live imaging

5x10^5^ J774A.1 macrophages were plated in a 10 mm Glass Bottom Culture 35 mm petri dish (MATEK corporation, P35G-0-14-C) and incubated 12–16 h at 37 °C and 5% CO_2_. The next day, cells were infected with mCherry-398 at an MOI≈10 as described above. 15 minutes prior to the indicated infection time points, cells were washed twice with PBS and incubated in DMEM with LysoSensor Green DND-189 (1μM; Molecular Probes, Invitrogen, L7535). The cells were then rinsed twice in PBS and were immediately analyzed by confocal microscopy. During image acquisition, the cells were maintained at 37 °C and 5% CO_2_ in a temperature-controlled CO_2_ chamber on the microscope.

### Transmission electron microscopy

Cells from BALF were centrifuged at 300 x g for 5 minutes and incubated in 500μl Pharm Lyse Buffer (BD Biosciences 555899) for 3 minutes at room temperature to lyse red blood cells. The cells were centrifugated and resuspended in PBS. Total cells number and viability were measured using Trypan Blue solution (Sigma T8154) and counted with TC20 automated cell counter (BioRad). At least 1x10^6^ cells were fixed in 2% paraformaldehyde/2.5% glutaraldehyde (Polysciences Inc., Warrington, PA) in 100 mM sodium cacodylate buffer pH 7.2 for 1 h at room temperature and subsequently incubated to 4 °C overnight. Samples were washed in sodium cacodylate buffer at room temperature and postfixed in 1% osmium tetroxide (Polysciences Inc.) for 1 h. The cells were then rinsed in distilled water, and bloc-stained for 1 h with 1% aqueous uranyl acetate (Ted Pella Inc., Redding, CA). Subsequently, the samples were rinsed in distilled water several times, dehydrated in a graded series of ethanol, and finally embedded in Eponate 12 resin (Ted Pella Inc.). Sections of 95 nm were cut with a Leica Ultracut UCT ultramicrotome (Leica Microsystems Inc., Bannockburn, IL), stained with uranyl acetate and lead citrate, and viewed on a JEOL 1200 EX transmission electron microscope (JEOL USA Inc., Peabody, MA) equipped with an AMT 8-megapixel digital camera and AMT Image Capture Engine V602 software (Advanced Microscopy Techniques, Woburn, MA). Images were processed using ImageJ software.

### Statistical analysis

Statistical analyses were performed using GraphPad Prism 8.0 (GraphPad Software Inc., La Jolla, CA). Datasets were analyzed by Mann–Whitney test, Welch’s t-test, one way ANOVA-test or two-way ANOVA-test, as indicated.

## Supporting information

S1 Fig *A*. *baumannii* clinical isolate 398 infects AMs *in vivo* and survives inside ACVs. (A) Quantification of total AMs (CD45+CD11c+SiglecF+CD11b-) and PMNs (CD45+CD11b+Ly6G+) in the BALF of mice infected for 3 h or 24 h with GFP-398 or GFP-19606 strains. (B) Bacterial burden quantification in the lung after BALF extraction of mice infected for 3 h or 24 h with GFP-398 or GFP-19606 strains. (C) Individual channels from confocal micrograph showed in the Fig 1D.
(TIF)

S2 Fig *A*. *baumannii* clinical isolate 398 survives inside large ACVs in AMs. (A) Representative confocal microscopy images of cells present in the BALF of infected mice 24 hpi. Cell nuclei were stained with DAPI (blue), *A baumannii* 19606 or 398 were detected by GFP fluorescence (green), and actin was labeled with Alexa Fluor 555 phalloidin (red). Scale bars: 10 μm. Insets (40 μm) are a higher magnification of the area indicated with a white box in the corresponding image. (B) Comparison of the percentage of AM with 398 or 19606 ACVs at 24 hpi. At least 200 infected cells were analyzed per strain. The results are expressed as mean ± standard error of the mean (SEM) of three independent experiments.
(TIF)

S3 Fig The number of 398 replicative centers increase during the infection. J774A.1 cells infected with GFP-398 were analyzed by confocal microscopy and percents of replicative centers were quantified at 4, 6 and 24 hpi. Replicative centers were defined as ACVs with at least a size three times larger than the median size of the vacuoles at 2 hpi. Results are expressed as mean ± SEM of three independent experiments. Statistical analyses were performed using one way ANOVA-test, *< 0.0429, **< 0.0078. At least 200 infected cells were analyzed per indicated time point.
(TIFF)

S4 Fig The ACV interacts with the early marker EEA1. (A) Single channel images from the inset micrograph shown in Fig 2A. Bars: 10 μm.
(TIF)

S5 Fig 398 and 19606 ACVs colocalize with the late marker LAMP1. (A) Single channel images of the inset micrograph shown in panel 2C. Bars: 10 μm. (B) J774A.1 macrophages were infected with strains GFP-398 or GFP-19606 and fixed 4 hpi. The samples were stained to observe cell nuclei (blue), GFP-*A*. *baumannii* (green) and LAMP1 (red). Representative confocal images of the infections are shown. White arrows indicate ACVs that colocalize with the marker LAMP1. Bars: 20 μm. Insets (40 μm) are a higher magnification of region indicated in the corresponding image with a white box. (C) Quantification of LAMP1+ 398 or 19606 ACVs. At least 200 infected cells were analyzed. The results are expressed as means ± SEM of three independent experiments.
(TIF)

S6 Fig *A*. *baumannii* intracellular replication is not cytotoxic. LDH activity in the supernatant of infected macrophages was measured at 24 hpi. Percentage of cytotoxicity was calculated as the activity of released LDH relative to total LDH activity. The mean ± S.D. for three independent experiments is shown. Statistical analysis was performed by two-way ANOVA-test, **** < 0.0001.
(TIF)

S7 Fig 398 ACV does not colocalize with the autophagic marker LC3. (A) Single channel images of the inset micrograph shown in panel 3A. Bars: 10 μm.
(TIF)

S8 Fig DQ-BSA green fluorescence in non-infected or *A*. *baumannii* infected cells. (A) Representative image of non-infected J774A.1 cells treated with DQ-BSA green. (B) Single channel images of the inset micrograph shown in Fig 4A. Bars: 5 μm.
(TIF)

S9 Fig Bafilomycin A1 treatment improves 398 replication. (A) J774A.1 macrophages were infected with GFP-398 and treated with the proton pump V-ATPase inhibitor bafilomycin A1. Total numbers of intracellular CFU were determined at different times pi in treated and non-treated cells. Statistical analyses were performed by two-way ANOVA-test, ** < 0.0021. (B) Representative images of cells infected with GFP-398 (green) and incubated with or without bafilomycin A1 at 6 hpi are shown. Cell nuclei were stained with DAPI (blue) and actin with Alexa Fluor 555 Phalloidin (red). Bars: 20 μm.
(TIF)

S10 Fig 19606 decreases the luminal pH of the ACV during infection. (A) J774A.1 cells infected with mCherry-19606 (red) were incubated with LysoSensor (green) 15 minutes before the indicated time points. Samples were analyzed by *in vivo* confocal microscopy. Bars: 20 μm. (B) Analysis of the Mean Fluorescence Intensity (MFI) signal of LysoSensor per ACV at different times pi. Dotted lines show the median. (C) Percentage of ACVs that colocalize with LysoSensor at 2, 4, and 6 hpi. Statistical analyses were performed using one way ANOVA-test, ****< 0.00001, *< 0.02. At least 200 infected cells were analyzed per indicated time point. Results are expressed as mean ± SEM of three independent experiments.
(TIF)

S11 Fig Growth of *A*. *baumannii* strains at different pH. Growth of 398 and 19606 strains in LB buffered at (A) pH 6 or (B) pH 7 was measured by OD_600_.
(TIF)

S12 Fig The replicative capacity of *A*. *baumannii* clinical isolates is related to growth fitness at acidic pH. (A) Intracellular replication of *A*. *baumannii* clinical isolates 378, 438, 647, 795 and 803 in J774A.1 macrophages determined by antibiotic protection assays. Growth of *A*. *baumannii* strains in LB buffered at (B) pH 5, (E) pH 6 or (F) pH 7 was measured by OD_600_. (C) Changes in culture pH during *A*. *baumannii* strains growth, determined by phenol red absorbance at 560 nm. (D) Concentration of ammonia in LB cultures of *A*. *baumannii* strains at 4, 5 and 6 h post-inoculation. Results are expressed as mean ± SEM of three independent experiments. ****< 0.0001, ***< 0.001, **< 0.01 and *< 0.05.
(TIF)

S1 Table Bacterial strains and plasmids. (DOCX)

## References

[1] Di Venanzio G, Flores-Mireles AL, Calix JJ, Haurat MF, Scott NE, Palmer LD, et al. Urinary tract colonization is enhanced by a plasmid that regulates uropathogenic Acinetobacter baumannii chromosomal genes. Nat Commun. 2019;10: 1100. doi: 10.1038/s41467-019-10706-y 31235751 PMC6591400

[2] Al Mubarak R, Roberts N, Mason RJ, Alper S, Chu HW. Comparison of pro- and anti-inflammatory responses in paired human primary airway epithelial cells and alveolar macrophages. Respir Res. 2018;19: 126. doi: 10.1186/s12931-018-0825-9 29940963 PMC6020222

[3] Bissonnette EY, Lauzon-Joset J-F, Debley JS, Ziegler SF. Cross-Talk Between Alveolar Macrophages and Lung Epithelial Cells is Essential to Maintain Lung Homeostasis. Front Immunol. 2020;11. doi: 10.3389/fimmu.2020.583042 33178214 PMC7593577

[4] Pires S, Parker D. Innate Immune Responses to Acinetobacter baumannii in the Airway. J Interf Cytokine Res. 2019;39: 441–449. doi: 10.1089/jir.2019.0008 31013462 PMC6909682

[5] Qiu H, KuoLee R, Harris G, Van Rooijen N, Patel GB, Chen W. Role of Macrophages in Early Host Resistance to Respiratory Acinetobacter baumannii Infection. PLoS One. 2012;7: e40019. doi: 10.1371/journal.pone.0040019 22768201 PMC3386929

[6] García-Patiño MG, García-Contreras R, Licona-Limón P. The Immune Response against Acinetobacter baumannii, an Emerging Pathogen in Nosocomial Infections. Front Immunol. 2017;8. doi: 10.3389/fimmu.2017.00441 28446911 PMC5388700

[7] Lee HH, Aslanyan L, Vidyasagar A, Brennan MB, Tauber MS, Carrillo-Sepulveda MA, et al. Depletion of Alveolar Macrophages Increases Pulmonary Neutrophil Infiltration, Tissue Damage, and Sepsis in a Murine Model of Acinetobacter baumannii Pneumonia. Torres VJ, editor. Infect Immun. 2020;88: 1–15. doi: 10.1128/IAI.00128-20 32366576 PMC7309625

[8] Rosales C, Uribe-Querol E. Phagocytosis: A Fundamental Process in Immunity. Biomed Res Int. 2017;2017: 1–18. doi: 10.1155/2017/9042851 28691037 PMC5485277

[9] Uribe-Querol E, Rosales C. Control of Phagocytosis by Microbial Pathogens. Front Immunol. 2017;8: 1–23. doi: 10.3389/fimmu.2017.01368 29114249 PMC5660709

[10] Lim JJ, Grinstein S, Roth Z. Diversity and Versatility of Phagocytosis: Roles in Innate Immunity, Tissue Remodeling, and Homeostasis. Front Cell Infect Microbiol. 2017;7: 1–12. doi: 10.3389/fcimb.2017.00191 28589095 PMC5440456

[11] Rabinovitch M. Professional and non-professional phagocytes: an introduction. Trends Cell Biol. 1995;5: 85–87. doi: 10.1016/s0962-8924(00)88955-2 14732160

[12] Kinchen JM, Ravichandran KS. Phagosome maturation: going through the acid test. Nat Rev Mol Cell Biol. 2008;9: 781–795. doi: 10.1038/nrm2515 18813294 PMC2908392

[13] Fountain A, Inpanathan S, Alves P, Verdawala MB, Botelho RJ. Phagosome maturation in macrophages: Eat, digest, adapt, and repeat. Adv Biol Regul. 2021;82: 100832. doi: 10.1016/j.jbior.2021.100832 34717137

[14] Lee H-J, Woo Y, Hahn T-W, Jung YM, Jung Y-J. Formation and Maturation of the Phagosome: A Key Mechanism in Innate Immunity against Intracellular Bacterial Infection. Microorganisms. 2020;8: 1298. doi: 10.3390/microorganisms8091298 32854338 PMC7564318

[15] Westman J, Walpole GFW, Kasper L, Xue BY, Elshafee O, Hube B, et al. Lysosome Fusion Maintains Phagosome Integrity during Fungal Infection. Cell Host Microbe. 2020;28: 798–812.e6. doi: 10.1016/j.chom.2020.09.004 33022213

[16] Nguyen JA, Yates RM. Better Together: Current Insights Into Phagosome-Lysosome Fusion. Front Immunol. 2021;12: 1–19. doi: 10.3389/fimmu.2021.636078 33717183 PMC7946854

[17] Jeschke A, Haas A. Sequential actions of phosphatidylinositol phosphates regulate phagosome-lysosome fusion. Gruenberg JE, editor. Mol Biol Cell. 2018;29: 452–465. doi: 10.1091/mbc.E17-07-0464 29237821 PMC6014173

[18] Banerjee S, Kane PM. Regulation of V-ATPase Activity and Organelle pH by Phosphatidylinositol Phosphate Lipids. Front Cell Dev Biol. 2020;8. doi: 10.3389/fcell.2020.00510 32656214 PMC7324685

[19] Kissing S, Hermsen C, Repnik U, Nesset CK, von Bargen K, Griffiths G, et al. Vacuolar ATPase in Phagosome-Lysosome Fusion. J Biol Chem. 2015;290: 14166–14180. doi: 10.1074/jbc.M114.628891 25903133 PMC4447986

[20] Westman J, Grinstein S. Determinants of Phagosomal pH During Host-Pathogen Interactions. Front Cell Dev Biol. 2021;0: 1781. doi: 10.3389/fcell.2020.624958 33505976 PMC7829662

[21] Dragotakes Q, Stouffer KM, Fu MS, Sella Y, Youn C, Yoon OI, et al. Macrophages use a bet-hedging strategy for antimicrobial activity in phagolysosomal acidification. J Clin Invest. 2020;130: 3805–3819. doi: 10.1172/JCI133938 32298242 PMC7346583

[22] Kellermann M, Scharte F, Hensel M. Manipulation of Host Cell Organelles by Intracellular Pathogens. Int J Mol Sci. 2021;22: 6484. doi: 10.3390/ijms22126484 34204285 PMC8235465

[23] Omotade TO, Roy CR. Manipulation of Host Cell Organelles by Intracellular Pathogens. Cossart P, Roy CR, Sansonetti P, editors. Microbiol Spectr. 2019;7: 6484. doi: 10.1128/microbiolspec.BAI-0022-2019 31025623 PMC11590418

[24] Celli J. The Intracellular Life Cycle of Brucella spp. Cossart P, Roy CR, Sansonetti P, editors. Microbiol Spectr. 2019;7: 289–313. doi: 10.1128/microbiolspec.BAI-0006-2019 30848234 PMC6448592

[25] Steiner B, Weber S, Hilbi H. Formation of the Legionella-containing vacuole: phosphoinositide conversion, GTPase modulation and ER dynamics. Int J Med Microbiol. 2018;308: 49–57. doi: 10.1016/j.ijmm.2017.08.004 28865995

[26] Gitsels A, Sanders N, Vanrompay D. Chlamydial Infection From Outside to Inside. Front Microbiol. 2019;10: 1–27. doi: 10.3389/fmicb.2019.02329 31649655 PMC6795091

[27] Queval CJ, Brosch R, Simeone R. The Macrophage: A Disputed Fortress in the Battle against Mycobacterium tuberculosis. Front Microbiol. 2017;8: 1–11. doi: 10.3389/fmicb.2017.02284 29218036 PMC5703847

[28] Brumell J. Salmonella redirects phagosomal maturation. Curr Opin Microbiol. 2004;7: 78–84. doi: 10.1016/j.mib.2003.12.005 15036145

[29] Steele-Mortimer O. The Salmonella-containing vacuole: moving with the times. Curr Opin Microbiol. 2008;11: 38–45. doi: 10.1016/j.mib.2008.01.002 18304858 PMC2577838

[30] van Schaik EJ, Chen C, Mertens K, Weber MM, Samuel JE. Molecular pathogenesis of the obligate intracellular bacterium Coxiella burnetii. Nat Rev Microbiol. 2013;11: 561–573. doi: 10.1038/nrmicro3049 23797173 PMC4134018

[31] Qiu J, Luo Z-Q. Legionella and Coxiella effectors: strength in diversity and activity. Nat Rev Microbiol. 2017;15: 591–605. doi: 10.1038/nrmicro.2017.67 28713154

[32] Rubio T, Gagné S, Debruyne C, Dias C, Cluzel C, Mongellaz D, et al. Incidence of an Intracellular Multiplication Niche among Acinetobacter baumannii Clinical Isolates. 2022 [cited 2 Feb 2022]. https://journals.asm.org/journal/msystems 10.1128/msystems.00488-21 PMC8805633 35103489

[33] Sycz G, Di Venanzio G, Distel JS, Sartorio MG, Le N-HH, Scott NE, et al. Modern Acinetobacter baumannii clinical isolates replicate inside spacious vacuoles and egress from macrophages. PLOS Pathog. 2021;17: e1009802. doi: 10.1371/journal.ppat.1009802 34370792 PMC8376066

[34] Sato Y, Unno Y, Miyazaki C, Ubagai T, Ono Y. Multidrug-resistant Acinetobacter baumannii resists reactive oxygen species and survives in macrophages. Sci Rep. 2019;9: 2–11. doi: 10.1038/s41598-019-53846-3 31767923 PMC6877552

[35] Riebisch AK, Mühlen S, Beer YY, Schmitz I. Autophagy—A Story of Bacteria Interfering with the Host Cell Degradation Machinery. Pathogens. 2021;10: 110. doi: 10.3390/pathogens10020110 33499114 PMC7911818

[36] Mao K, Klionsky DJ. Xenophagy: A battlefield between host and microbe, and a possible avenue for cancer treatment. Autophagy. 2017;13: 223–224. doi: 10.1080/15548627.2016.1267075 28026986 PMC5324837

[37] Sharma V, Verma S, Seranova E, Sarkar S, Kumar D. Selective Autophagy and Xenophagy in Infection and Disease. Front Cell Dev Biol. 2018;6: 1–17. doi: 10.3389/fcell.2018.00147 30483501 PMC6243101

[38] Grijmans BJM, van der Kooij SB, Varela M, Meijer AH. LAPped in Proof: LC3-Associated Phagocytosis and the Arms Race Against Bacterial Pathogens. Front Cell Infect Microbiol. 2022;11: 1–14. doi: 10.3389/fcimb.2021.809121 35047422 PMC8762105

[39] Mansilla Pareja ME, Bongiovanni A, Lafont F, Colombo MI. Alterations of the Coxiella burnetii Replicative Vacuole Membrane Integrity and Interplay with the Autophagy Pathway. Front Cell Infect Microbiol. 2017;7: 1–17. doi: 10.3389/fcimb.2017.00112 28484683 PMC5401879

[40] Winchell CG, Graham JG, Kurten RC, Voth DE. Coxiella burnetii Type IV Secretion-Dependent Recruitment of Macrophage Autophagosomes. Roy CR, editor. Infect Immun. 2014;82: 2229–2238. doi: 10.1128/IAI.01236-13 24643534 PMC4019161

[41] Fedrigo GV, Campoy EM, Di Venanzio G, Colombo MI, García Véscovi E. Serratia marcescens Is Able to Survive and Proliferate in Autophagic-Like Vacuoles inside Non-Phagocytic Cells. May RC, editor. PLoS One. 2011;6: 15. doi: 10.1371/journal.pone.0024054 21901159 PMC3162031

[42] Omotade TO, Roy CR. Legionella pneumophila Excludes Autophagy Adaptors from the Ubiquitin-Labeled Vacuole in Which It Resides. Brodsky IE, editor. Infect Immun. 2020;88. doi: 10.1128/IAI.00793-19 32482642 PMC7375760

[43] Strong EJ, Wang J, Ng TW, Porcelli SA, Lee S. Mycobacterium tuberculosis PPE51 Inhibits Autophagy by Suppressing Toll-Like Receptor 2-Dependent Signaling. Siegrist MS, editor. MBio. 2022;13. doi: 10.1128/mbio.02974-21 35467412 PMC9239179

[44] Ambrosi C, Scribano D, Sarshar M, Zagaglia C, Singer BB, Palamara AT. Acinetobacter baumannii Targets Human Carcinoembryonic Antigen-Related Cell Adhesion Molecules (CEACAMs) for Invasion of Pneumocytes. Langelier C, editor. mSystems. 2020;5: 1–17. doi: 10.1128/mSystems.00604-20 33361319 PMC7762790

[45] Glover JS, Browning BD, Ticer TD, Engevik AC, Engevik MA. Acinetobacter calcoaceticus is Well Adapted to Withstand Intestinal Stressors and Modulate the Gut Epithelium. Front Physiol. 2022;13: 1–13. doi: 10.3389/fphys.2022.880024 35685287 PMC9170955

[46] Peleg AY, de Breij A, Adams MD, Cerqueira GM, Mocali S, Galardini M, et al. The Success of Acinetobacter Species; Genetic, Metabolic and Virulence Attributes. de Crécy-Lagard V, editor. PLoS One. 2012;7: e46984. doi: 10.1371/journal.pone.0046984 23144699 PMC3483291

[47] Cook AM, Fewson CA. Role of cabohydrates in the metabolism of Acinetobacter calcoaceticus. Biochim Biophys Acta—Gen Subj. 1973;320: 214–216. doi: 10.1016/0304-4165(73)90181-5 4748364

[48] Actis LA, Smoot JC, Barancin CE, Findlay RH. Comparison of differential plating media and two chromatography techniques for the detection of histamine production in bacteria. J Microbiol Methods. 1999;39: 79–90. doi: 10.1016/s0167-7012(99)00099-8 10579509

[49] Gu H, Zeng X, Peng L, Xiang C, Zhou Y, Zhang X, et al. Vaccination induces rapid protection against bacterial pneumonia via training alveolar macrophage in mice. Elife. 2021;10: 1–19. doi: 10.7554/eLife.69951 34544549 PMC8455131

[50] Tsay T-B, Chang W-H, Hsu C-M, Chen L-W. Mechanical ventilation enhances Acinetobacter baumannii-induced lung injury through JNK pathways. Respir Res. 2021;22: 159. doi: 10.1186/s12931-021-01739-3 34022899 PMC8140754

[51] Huang L, Nazarova E V., Tan S, Liu Y, Russell DG. Growth of Mycobacterium tuberculosis in vivo segregates with host macrophage metabolism and ontogeny. J Exp Med. 2018;215: 1135–1152. doi: 10.1084/jem.20172020 29500179 PMC5881470

[52] Cohen SB, Gern BH, Delahaye JL, Adams KN, Plumlee CR, Winkler JK, et al. Alveolar Macrophages Provide an Early Mycobacterium tuberculosis Niche and Initiate Dissemination. Cell Host Microbe. 2018;24: 439–446.e4. doi: 10.1016/j.chom.2018.08.001 30146391 PMC6152889

[53] Lacoma A, Cano V, Moranta D, Regueiro V, Domínguez-Villanueva D, Laabei M, et al. Investigating intracellular persistence of Staphylococcus aureus within a murine alveolar macrophage cell line. Virulence. 2017;8: 1761–1775. doi: 10.1080/21505594.2017.1361089 28762868 PMC5810471

[54] Khweek AA, Amer A. Replication of Legionella Pneumophila in Human Cells: Why are We Susceptible? Front Microbiol. 2010;1: 1–8. doi: 10.3389/fmicb.2010.00133 21687775 PMC3109522

[55] Parra-Millán R, Guerrero-Gómez D, Ayerbe-Algaba R, Pachón-Ibáñez ME, Miranda-Vizuete A, Pachón J, et al. Intracellular Trafficking and Persistence of Acinetobacter baumannii Requires Transcription Factor EB. D’Orazio SEF, editor. mSphere. 2018;3: 1–14. doi: 10.1128/mSphere.00106-18 29600279 PMC5874439

[56] Spiering D, Hodgson L. Dynamics of the Rho-family small GTPases in actin regulation and motility. Cell Adh Migr. 2011;5: 170–180. doi: 10.4161/cam.5.2.14403 21178402 PMC3084983

[57] Wang Y, Zhang K, Shi X, Wang C, Wang F, Fan J, et al. Critical role of bacterial isochorismatase in the autophagic process induced by Acinetobacter baumannii in mammalian cells. FASEB J. 2016;30: 3563–3577. doi: 10.1096/fj.201500019R 27432399 PMC5024702

[58] Zhao J, Beyrakhova K, Liu Y, Alvarez CP, Bueler SA, Xu L, et al. Molecular basis for the binding and modulation of V-ATPase by a bacterial effector protein. PLOS Pathog. 2017;13: e1006394. doi: 10.1371/journal.ppat.1006394 28570695 PMC5469503

[59] Wong D, Bach H, Sun J, Hmama Z, Av-Gay Y. Mycobacterium tuberculosis protein tyrosine phosphatase (PtpA) excludes host vacuolar-H+-ATPase to inhibit phagosome acidification. Proc Natl Acad Sci U S A. 2011;108: 19371–6. doi: 10.1073/pnas.1109201108 22087003 PMC3228452

[60] Du J, Reeves AZ, Klein JA, Twedt DJ, Knodler LA, Lesser CF. The type III secretion system apparatus determines the intracellular niche of bacterial pathogens. Proc Natl Acad Sci. 2016;113: 4794–4799. doi: 10.1073/pnas.1520699113 27078095 PMC4855615

[61] Hayek I, Berens C, Lührmann A. Modulation of host cell metabolism by T4SS-encoding intracellular pathogens. Curr Opin Microbiol. 2019;47: 59–65. doi: 10.1016/j.mib.2018.11.010 30640035

[62] Weber BS, Kinsella RL, Harding CM, Feldman MF. The Secrets of Acinetobacter Secretion. Trends Microbiol. 2017;25: 532–545. doi: 10.1016/j.tim.2017.01.005 28216293 PMC5474180

[63] Hart PD, Young MR. Ammonium chloride, an inhibitor of phagosome-lysosome fusion in macrophages, concurrently induces phagosome-endosome fusion, and opens a novel pathway: studies of a pathogenic mycobacterium and a nonpathogenic yeast. J Exp Med. 1991;174: 881–889. doi: 10.1084/jem.174.4.881 1919441 PMC2118961

[64] Hart PD, Young MR. Effects of nitrogenous bases on macrophage lysosomal movements and phagosome-lysosome fusion. In: van Furth R, editor. Mononuclear Phagocytes. Dordrecht: Springer Netherlands; 1985. pp. 479–485. doi: 10.1007/978-94-009-5020-7_50

[65] Gordon AH, D’Arcy Hart P, Young MR. Ammonia inhibits phagosome–lysosome fusion in macrophages. Nature. 1980;286: 79–80. doi: 10.1038/286079a0 6993961

[66] Fu MS, Coelho C, De Leon-Rodriguez CM, Rossi DCP, Camacho E, Jung EH, et al. Cryptococcus neoformans urease affects the outcome of intracellular pathogenesis by modulating phagolysosomal pH. May RC, editor. PLOS Pathog. 2018;14: e1007144. doi: 10.1371/journal.ppat.1007144 29906292 PMC6021110

[67] Schwartz JT. Role of urease in megasome formation and Helicobacter pylori survival in macrophages. J Leukoc Biol. 2006;79: 1214–1225. doi: 10.1189/jlb.0106030 16543403 PMC1868427

[68] Gouzy A, Larrouy-Maumus G, Bottai D, Levillain F, Dumas A, Wallach JB, et al. Mycobacterium tuberculosis Exploits Asparagine to Assimilate Nitrogen and Resist Acid Stress during Infection. Behr MA, editor. PLoS Pathog. 2014;10: e1003928. doi: 10.1371/journal.ppat.1003928 24586151 PMC3930563

[69] Dikshit N, Kale SD, Khameneh HJ, Balamuralidhar V, Tang CY, Kumar P, et al. NLRP3 inflammasome pathway has a critical role in the host immunity against clinically relevant Acinetobacter baumannii pulmonary infection. Mucosal Immunol. 2017 [cited 5 Sep 2017]. doi: 10.1038/mi.2017.50 28612844

[70] Li F-J, Starrs L, Mathur A, Ishii H, Man SM, Burgio G. Differential activation of NLRP3 inflammasome by Acinetobacter baumannii strains. Yenugu S, editor. PLoS One. 2022;17: e0277019. doi: 10.1371/journal.pone.0277019 36318583 PMC9624416

[71] McClure EE, Chávez ASO, Shaw DK, Carlyon JA, Ganta RR, Noh SM, et al. Engineering of obligate intracellular bacteria: progress, challenges and paradigms. Nat Rev Microbiol. 2017;15: 544–558. doi: 10.1038/nrmicro.2017.59 28626230 PMC5557331

[72] Hazen JE, Di Venanzio G, Hultgren SJ, Feldman MF. Catheterization of mice triggers resurgent urinary tract infection seeded by a bladder reservoir of Acinetobacter baumannii. Sci Transl Med. 2023;15: eabn8134. doi: 10.1126/scitranslmed.abn8134 36630484 PMC10464790

[73] Rovati L, Fabbri P, Ferrari L, Pilati F. Plastic Optical Fiber pH Sensor Using a Sol-Gel Sensing Matrix. Fiber Optic Sensors. InTech; 2012. doi: 10.5772/26517

[74] Paye MF, Rose HB, Robbins JM, Yunda DA, Cho S, Bommarius AS. A high-throughput pH-based colorimetric assay: application focus on alpha/beta hydrolases. Anal Biochem. 2018;549: 80–90. doi: 10.1016/j.ab.2018.03.009 29551670

[75] Held P. Using Phenol Red to Assess pH in Tissue Culture Media Using. BioTek Instruments, Inc. 2018; 7.

