# Supplementary figures and images for: Replicative Acinetobacter baumannii strains interfere with phagosomal maturation by modulating the vacuolar pH
Source: PLoS Pathog. 2023 Jun 9;19(6):e1011173. doi: 10.1371/journal.ppat.1011173 (PMC10286980; doi:10.1371/journal.ppat.1011173)

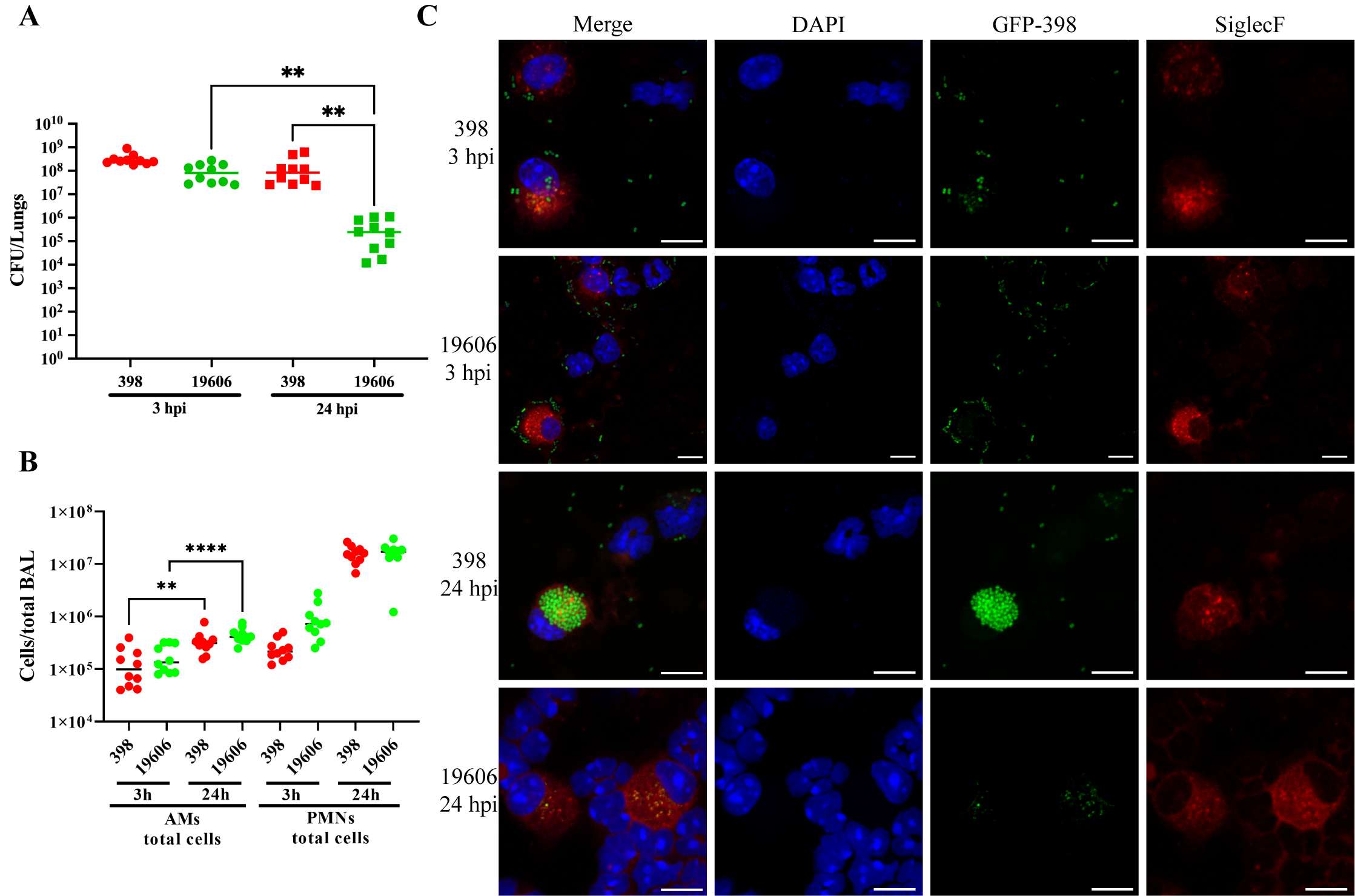

Supplement: S1 Fig — (A) Quantification of total AMs (CD45+CD11c+SiglecF+CD11b-) and PMNs (CD45+CD11b+Ly6G+) in the BALF of mice infected for 3 h or 24 h with GFP-398 or GFP-19606 strains. (B) Bacterial burden quantification in the lung after BALF extraction of mice infected for 3 h or 24 h with GFP-398 or GFP-19606 strains. (C) Individual channels from confocal micrograph showed in the Fig 1D. (TIF) [file ppat.1011173.s001.tif]

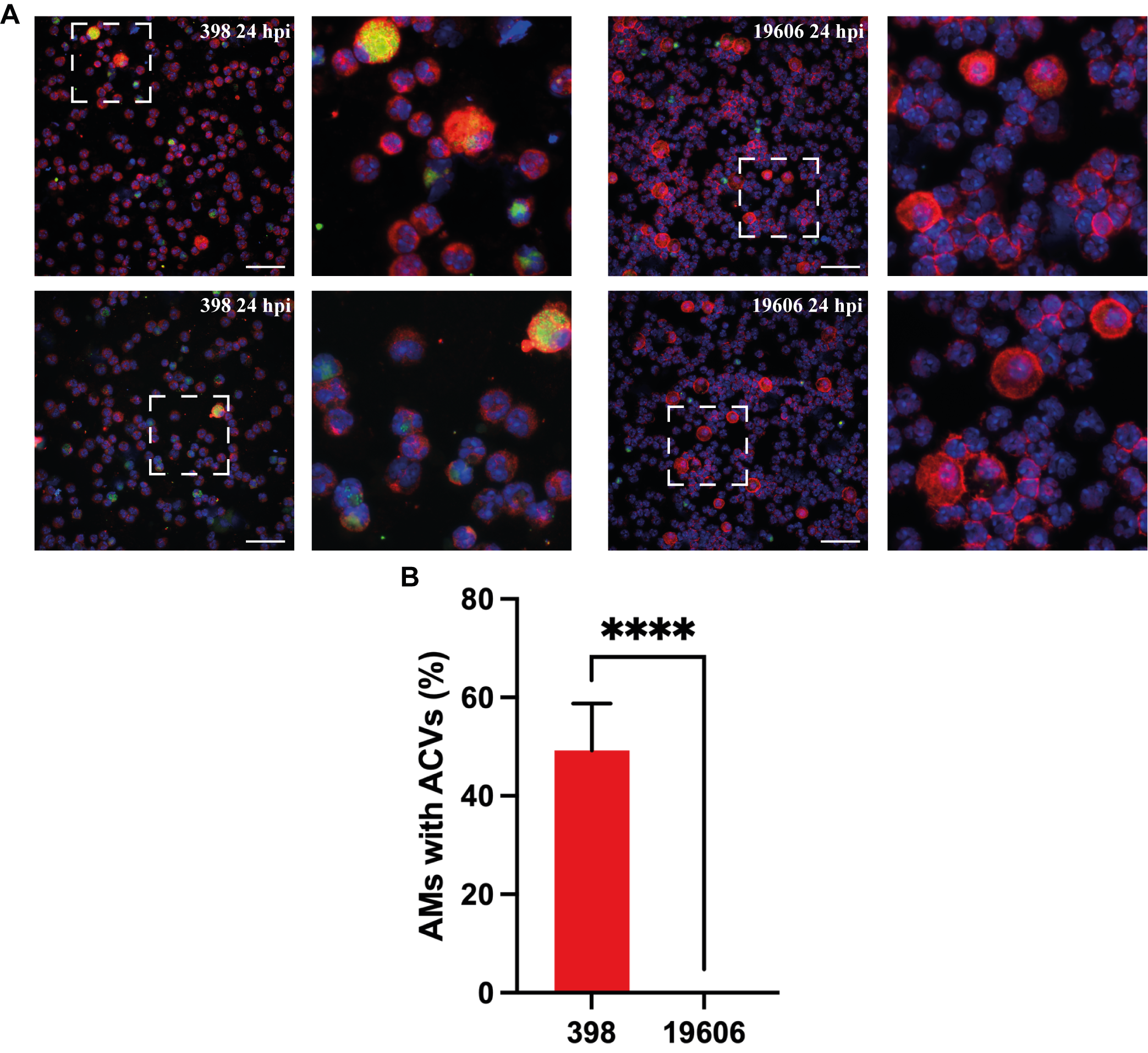

Supplement: S2 Fig — (A) Representative confocal microscopy images of cells present in the BALF of infected mice 24 hpi. Cell nuclei were stained with DAPI (blue), A baumannii 19606 or 398 were detected by GFP fluorescence (green), and actin was labeled with Alexa Fluor 555 phalloidin (red). Scale bars: 10 μm. Insets (40 μm) are a higher magnification of the area indicated with a white box in the corresponding image. (B) Comparison of the percentage of AM with 398 or 19606 ACVs at 24 hpi. At least 200 infected cells were analyzed per strain. The results are expressed as mean ± standard error of the mean (SEM) of three independent experiments. (TIF) [file ppat.1011173.s002.tif]

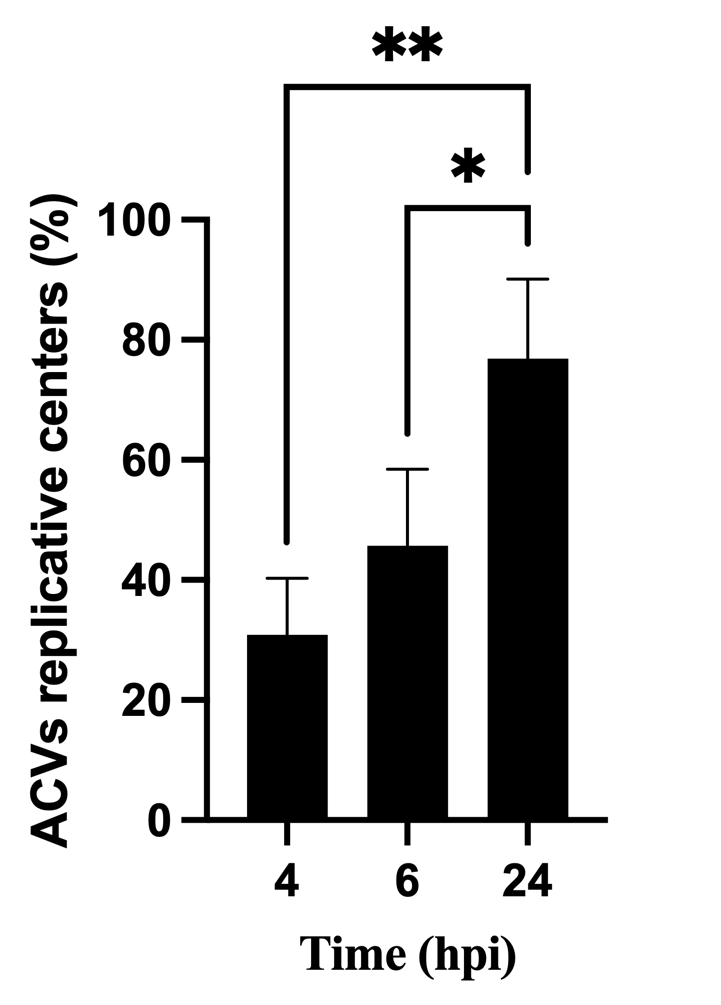

Supplement: S3 Fig — J774A.1 cells infected with GFP-398 were analyzed by confocal microscopy and percents of replicative centers were quantified at 4, 6 and 24 hpi. Replicative centers were defined as ACVs with at least a size three times larger than the median size of the vacuoles at 2 hpi. Results are expressed as mean ± SEM of three independent experiments. Statistical analyses were performed using one way ANOVA-test, *< 0.0429, **< 0.0078. At least 200 infected cells were analyzed per indicated time point. (TIFF) [file ppat.1011173.s003.tiff]

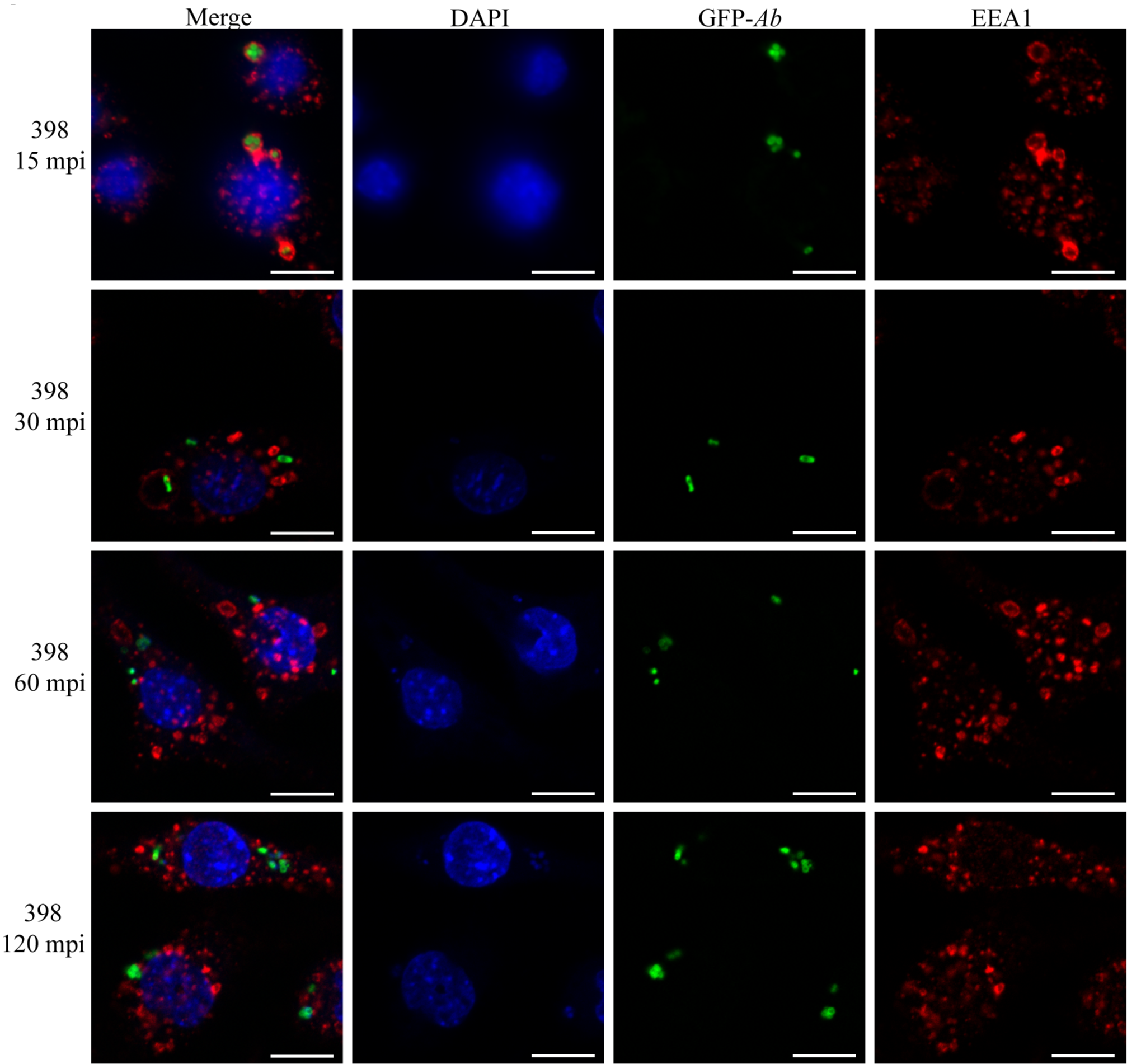

Supplement: S4 Fig — (A) Single channel images from the inset micrograph shown in Fig 2A. Bars: 10 μm. (TIF) [file ppat.1011173.s004.tif]

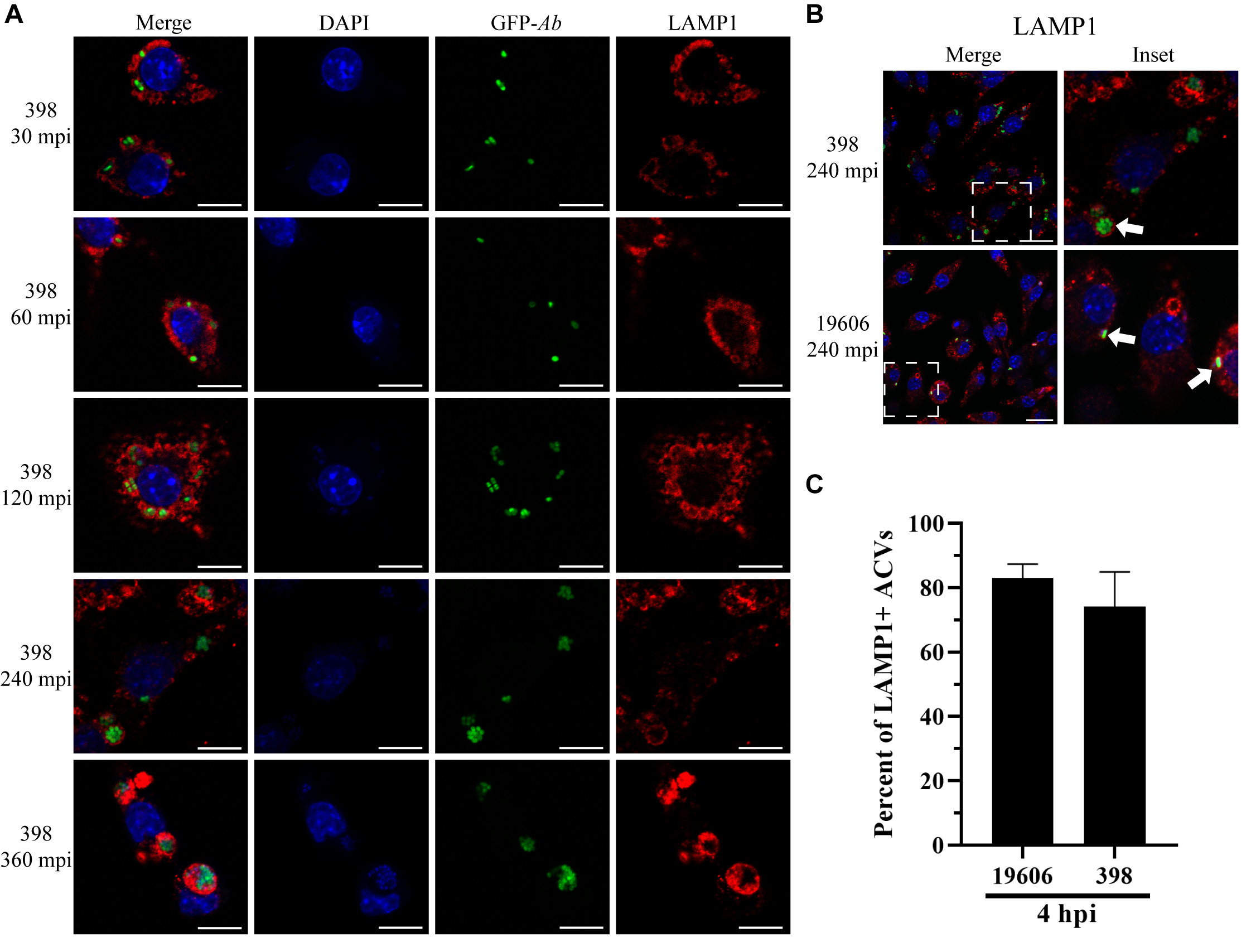

Supplement: S5 Fig — (A) Single channel images of the inset micrograph shown in panel 2C. Bars: 10 μm. (B) J774A.1 macrophages were infected with strains GFP-398 or GFP-19606 and fixed 4 hpi. The samples were stained to observe cell nuclei (blue), GFP-A. baumannii (green) and LAMP1 (red). Representative confocal images of the infections are shown. White arrows indicate ACVs that colocalize with the marker LAMP1. Bars: 20 μm. Insets (40 μm) are a higher magnification of region indicated in the corresponding image with a white box. (C) Quantification of LAMP1+ 398 or 19606 ACVs. At least 200 infected cells were analyzed. The results are expressed as means ± SEM of three independent experiments. (TIF) [file ppat.1011173.s005.tif]

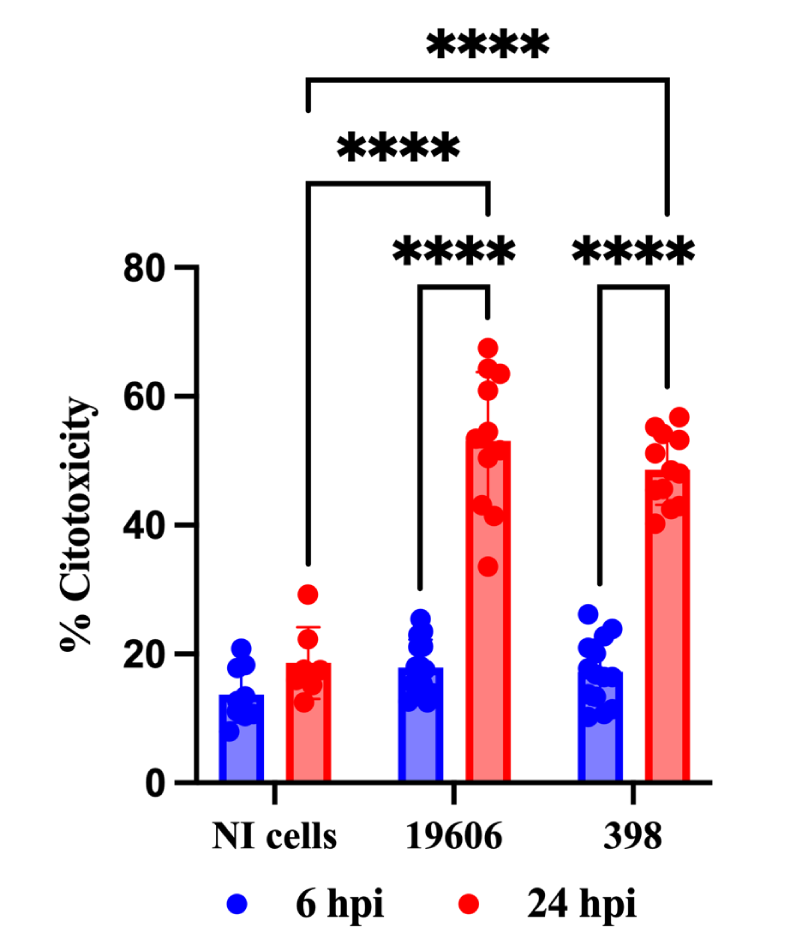

Supplement: S6 Fig — LDH activity in the supernatant of infected macrophages was measured at 24 hpi. Percentage of cytotoxicity was calculated as the activity of released LDH relative to total LDH activity. The mean ± S.D. for three independent experiments is shown. Statistical analysis was performed by two-way ANOVA-test, **** < 0.0001. (TIF) [file ppat.1011173.s006.tif]

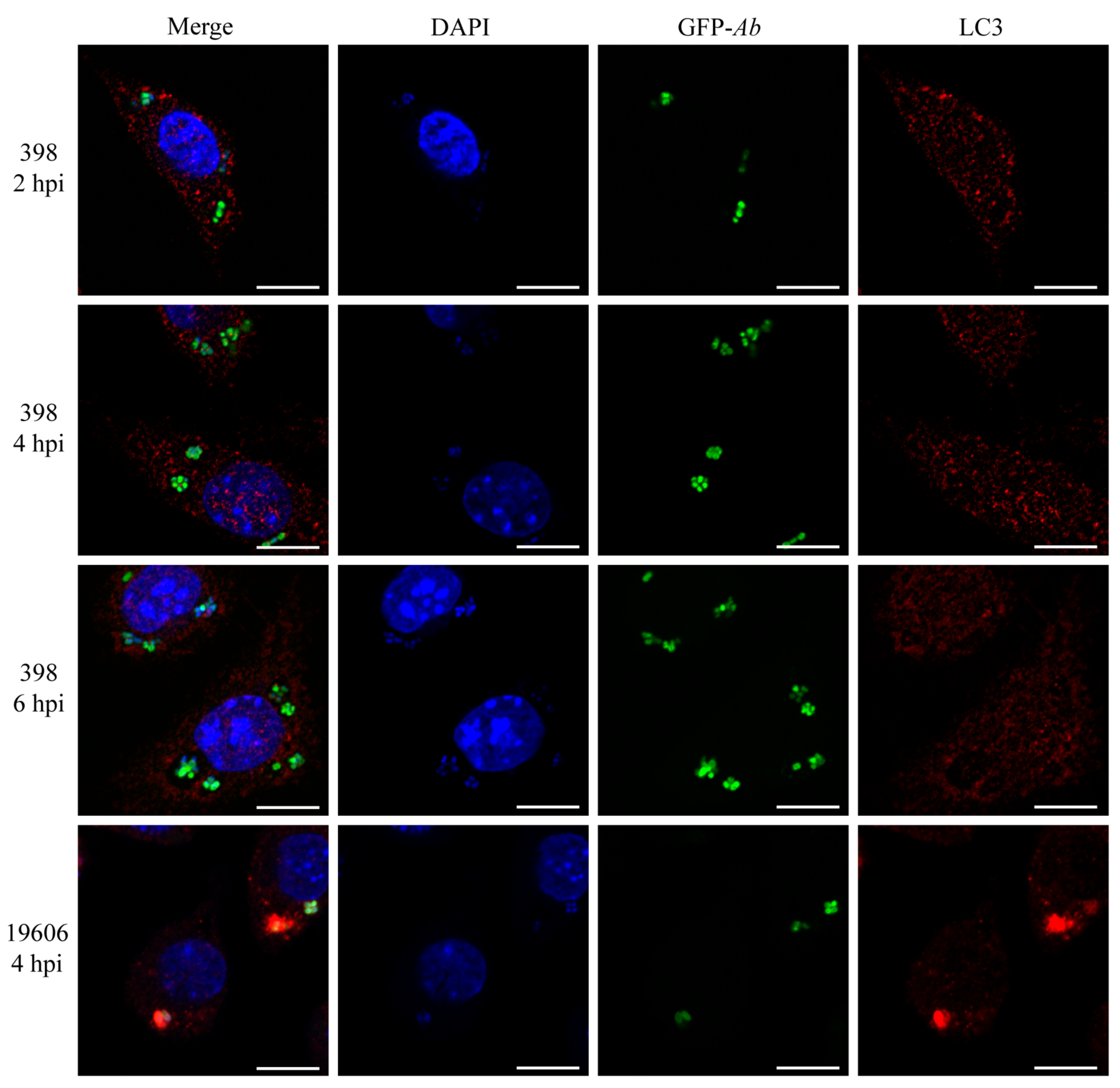

Supplement: S7 Fig — (A) Single channel images of the inset micrograph shown in panel 3A. Bars: 10 μm. (TIF) [file ppat.1011173.s007.tif]

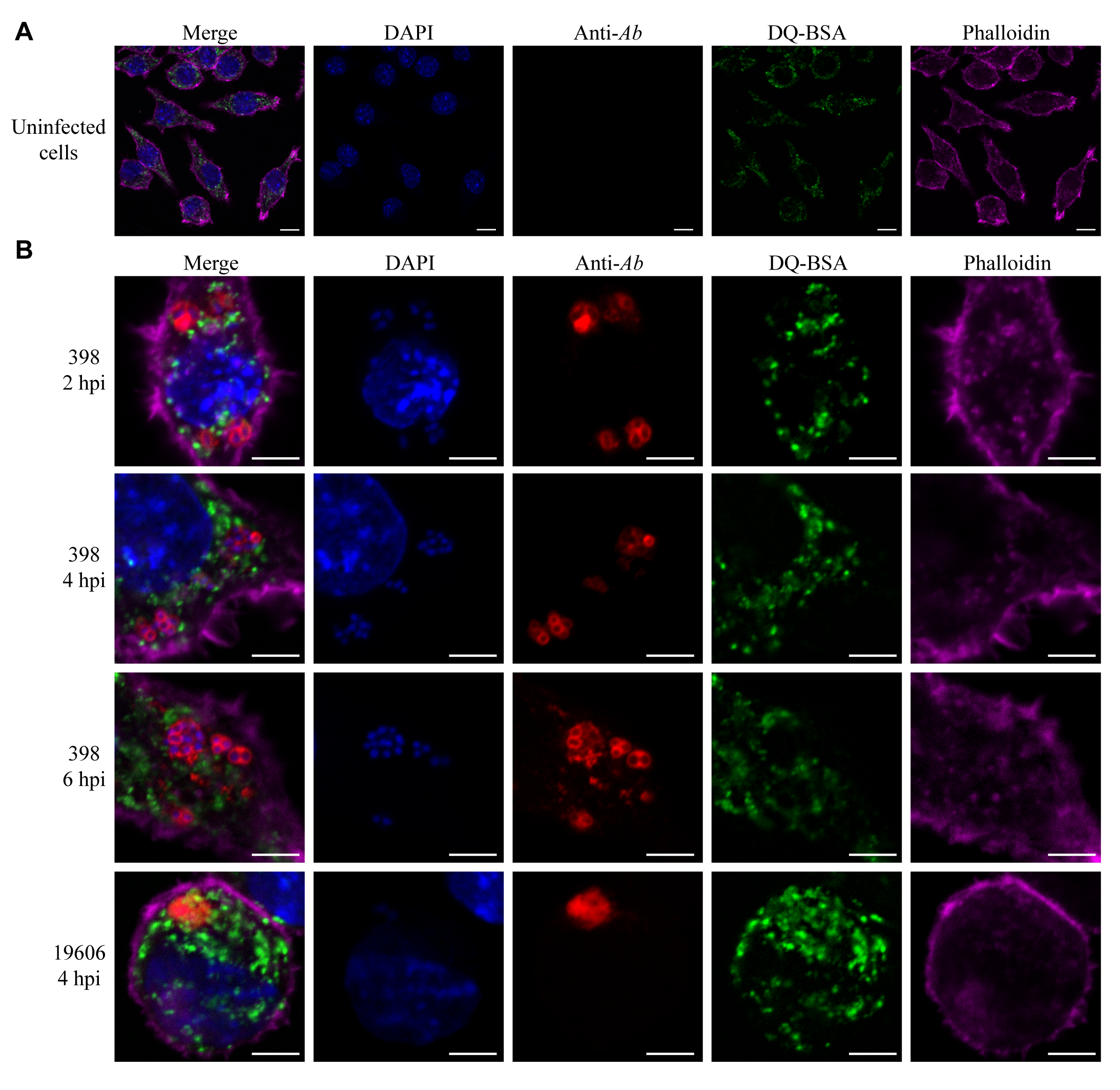

Supplement: S8 Fig — (A) Representative image of non-infected J774A.1 cells treated with DQ-BSA green. (B) Single channel images of the inset micrograph shown in Fig 4A. Bars: 5 μm. (TIF) [file ppat.1011173.s008.tif]

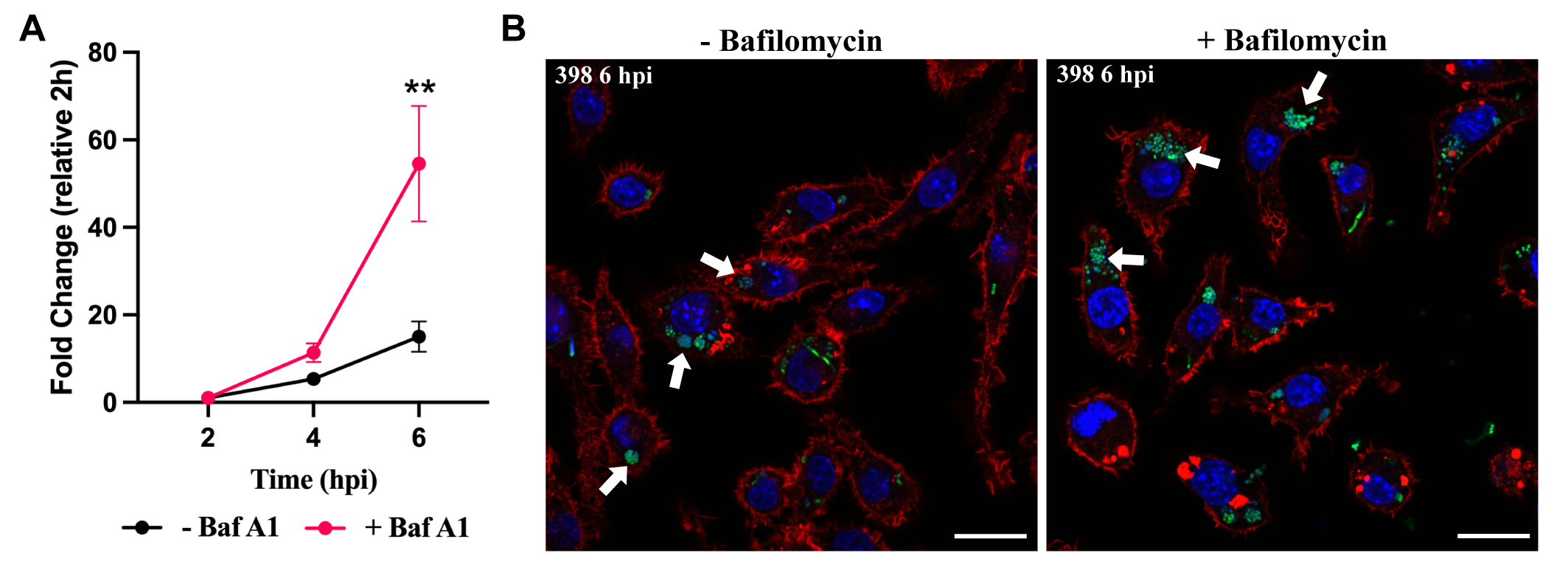

Supplement: S9 Fig — (A) J774A.1 macrophages were infected with GFP-398 and treated with the proton pump V-ATPase inhibitor bafilomycin A1. Total numbers of intracellular CFU were determined at different times pi in treated and non-treated cells. Statistical analyses were performed by two-way ANOVA-test, ** < 0.0021. (B) Representative images of cells infected with GFP-398 (green) and incubated with or without bafilomycin A1 at 6 hpi are shown. Cell nuclei were stained with DAPI (blue) and actin with Alexa Fluor 555 Phalloidin (red). Bars: 20 μm. (TIF) [file ppat.1011173.s009.tif]

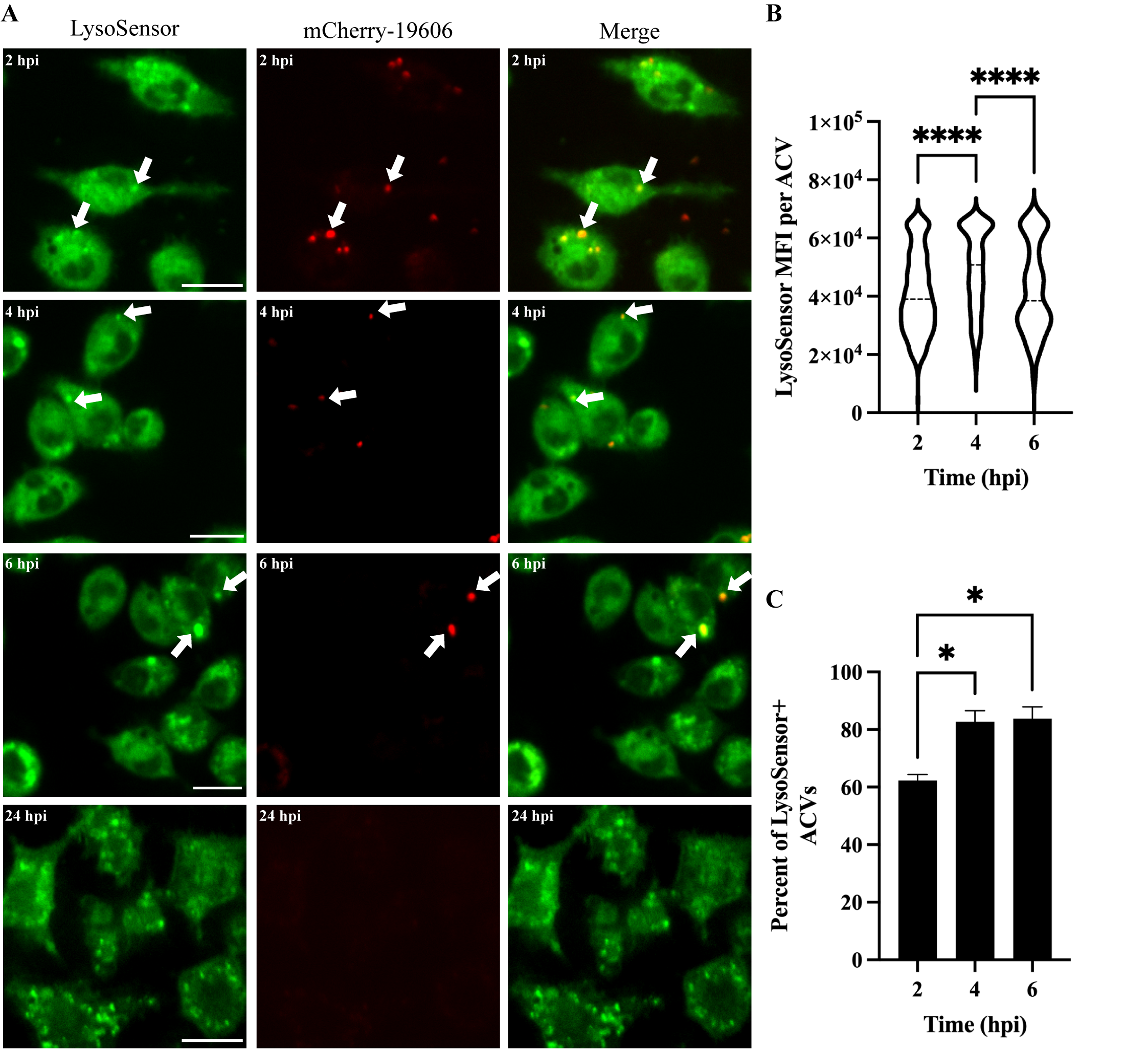

Supplement: S10 Fig — (A) J774A.1 cells infected with mCherry-19606 (red) were incubated with LysoSensor (green) 15 minutes before the indicated time points. Samples were analyzed by in vivo confocal microscopy. Bars: 20 μm. (B) Analysis of the Mean Fluorescence Intensity (MFI) signal of LysoSensor per ACV at different times pi. Dotted lines show the median. (C) Percentage of ACVs that colocalize with LysoSensor at 2, 4, and 6 hpi. Statistical analyses were performed using one way ANOVA-test, ****< 0.00001, *< 0.02. At least 200 infected cells were analyzed per indicated time point. Results are expressed as mean ± SEM of three independent experiments. (TIF) [file ppat.1011173.s010.tif]

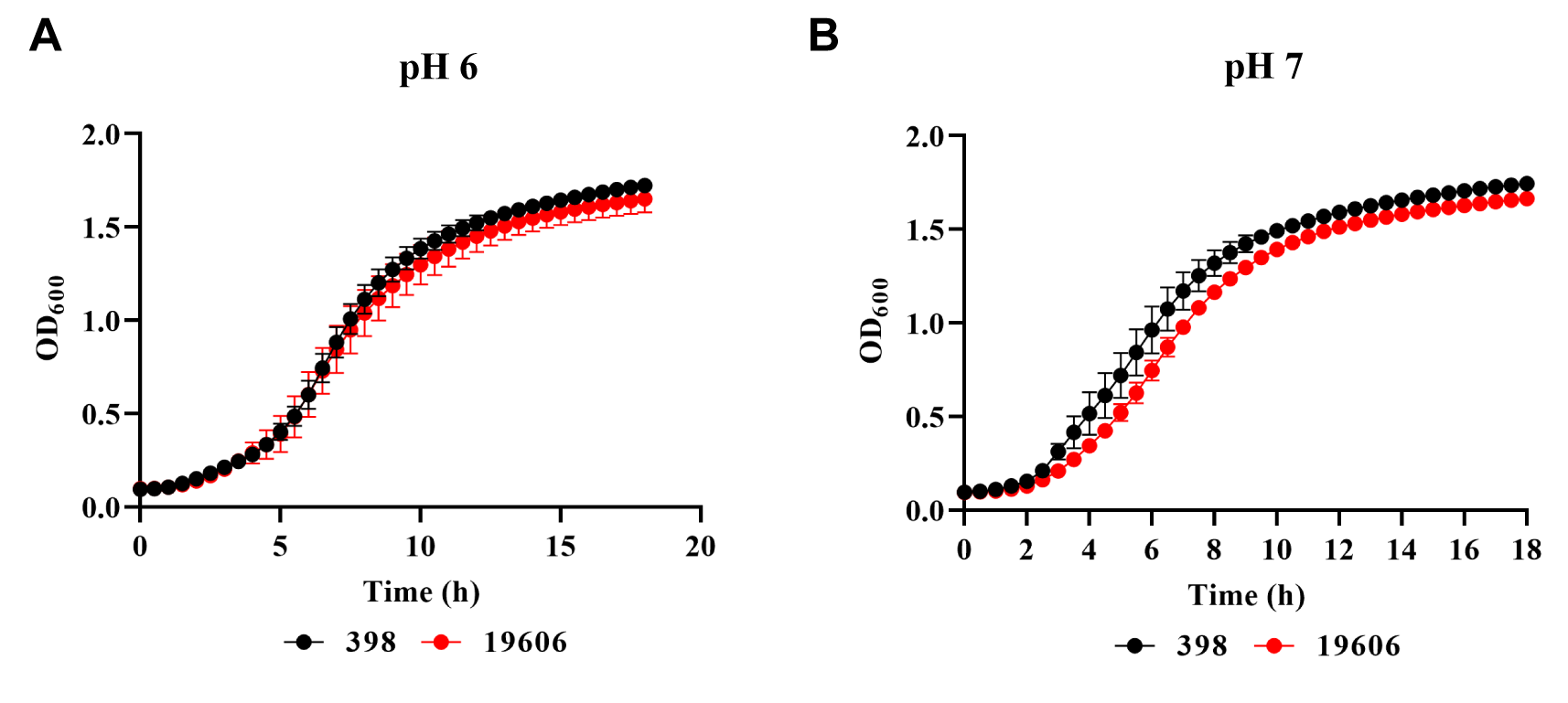

Supplement: S11 Fig — Growth of 398 and 19606 strains in LB buffered at (A) pH 6 or (B) pH 7 was measured by OD600. (TIF) [file ppat.1011173.s011.tif]

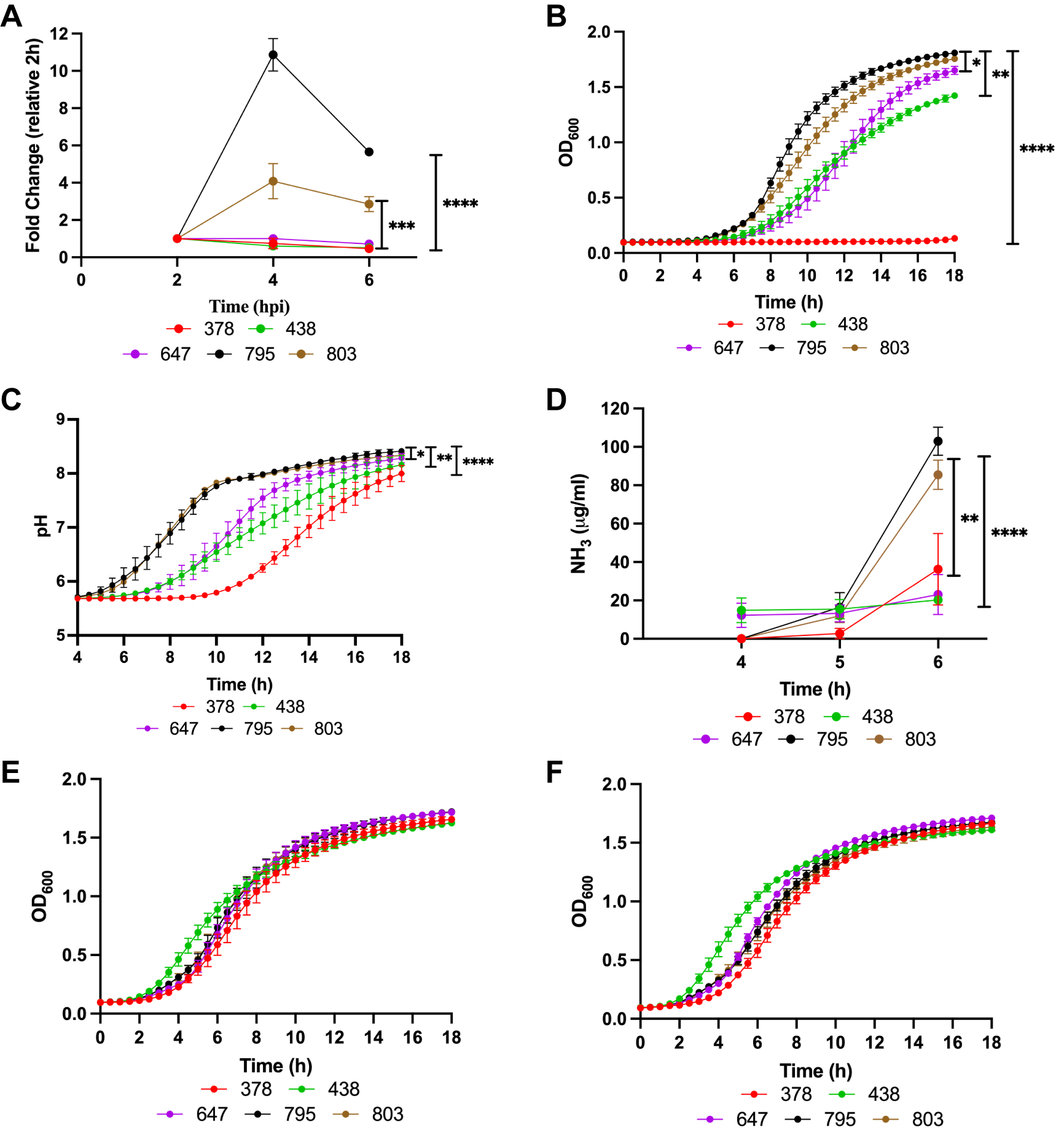

Supplement: S12 Fig — (A) Intracellular replication of A. baumannii clinical isolates 378, 438, 647, 795 and 803 in J774A.1 macrophages determined by antibiotic protection assays. Growth of A. baumannii strains in LB buffered at (B) pH 5, (E) pH 6 or (F) pH 7 was measured by OD600. (C) Changes in culture pH during A. baumannii strains growth, determined by phenol red absorbance at 560 nm. (D) Concentration of ammonia in LB cultures of A. baumannii strains at 4, 5 and 6 h post-inoculation. Results are expressed as mean ± SEM of three independent experiments. ****< 0.0001, ***< 0.001, **< 0.01 and *< 0.05. (TIF) [file ppat.1011173.s012.tif]
