# Supplementary material for: Replicative Acinetobacter baumannii strains interfere with phagosomal maturation by modulating the vacuolar pH
Source: PLoS Pathog. 2023 Jun 9;19(6):e1011173. doi: 10.1371/journal.ppat.1011173 (PMC10286980; doi:10.1371/journal.ppat.1011173)
Supplement: S1 Table — (DOCX) [file ppat.1011173.s013.docx]

**Table S1. Bacterial strains and plasmids.**

| **Bacterial strain** | **Description** | **Reference** |
| --- | --- | --- |
| ***Acinetobacter baumannii*** | **-** | **-** |
| 19606 | Urinary tract infection isolate | [1] |
| 398 | Urinary tract infection isolate | IHMA Labs. -2019 |
| 367 | Urinary tract infection isolate | Enthasis Therapeutics - 2013 |
| 378 | Respiratory infection isolate | JMI lab - 2018 |
| 438 | Respiratory infection isolate | Emory - before 2019 |
| 647 | Respiratory infection isolate | Emory - before 2019 |
| 795 | Respiratory infection isolate | Iran - 2019 |
| 803 | Respiratory infection isolate | Iran - 2019 |
| 19606 GFP | GFP-expressing transconjugant | [2] |
| 19606 mCherry | mCherry expressing strain | This work |
| 398 GFP | GFP-expressing transconjugant | [2] |
| 398 mCherry | mCherry-expressing transconjugant | This work. |
| **Plasmid** | - | - |
| pBAV1k-t5-gfp | Published vector, kanamycin resistance marker | [3] |
| pWH-mcherry | promotor promlac and mcherry gene from pBAV1k-t5-mCherry in pWH1266 | This work |
| pUC18T-mini-Tn7T-Zeo-GFP | promotor promlac and gfp gene from pBAV1k-t5-gfp on pUC18T-mini-Tn7T-Zeo backbone, zeocin resistance marker [2] | [2] |
| pUC18T-mini-Tn7T-Zeo-mCherry | promotor promlac and mcherry gene from pBAV1k-t5-mCherry on pUC18T-mini-Tn7T-Zeo backbone, zeocin resistance marker | This work. |

References

1. Hugh R, Reese R. Designation of the type strain for Bacterium anitratum Schaub and Hauber 1948. Int J Syst Bacteriol. 1967;17: 245–254. doi:10.1099/00207713-17-3-245

2. Sycz G, Di Venanzio G, Distel JS, Sartorio MG, Le N-HH, Scott NE, et al. Modern Acinetobacter baumannii clinical isolates replicate inside spacious vacuoles and egress from macrophages. PLOS Pathog. 2021;17: e1009802. doi:10.1371/JOURNAL.PPAT.1009802

3. Bryksin A V., Matsumura I. Rational Design of a Plasmid Origin That Replicates Efficiently in Both Gram-Positive and Gram-Negative Bacteria. Mokrousov I, editor. PLoS One. 2010;5: e13244. doi:10.1371/journal.pone.0013244
